# How to Compute Atomistic Insight in DFT Clusters: The REG-IQA Approach

**DOI:** 10.1021/acs.jcim.3c00404

**Published:** 2023-07-10

**Authors:** Fabio Falcioni, Paul L. A. Popelier

**Affiliations:** Department of Chemistry, University of Manchester, Oxford Road, Manchester M13 9PL, Great Britain

## Abstract

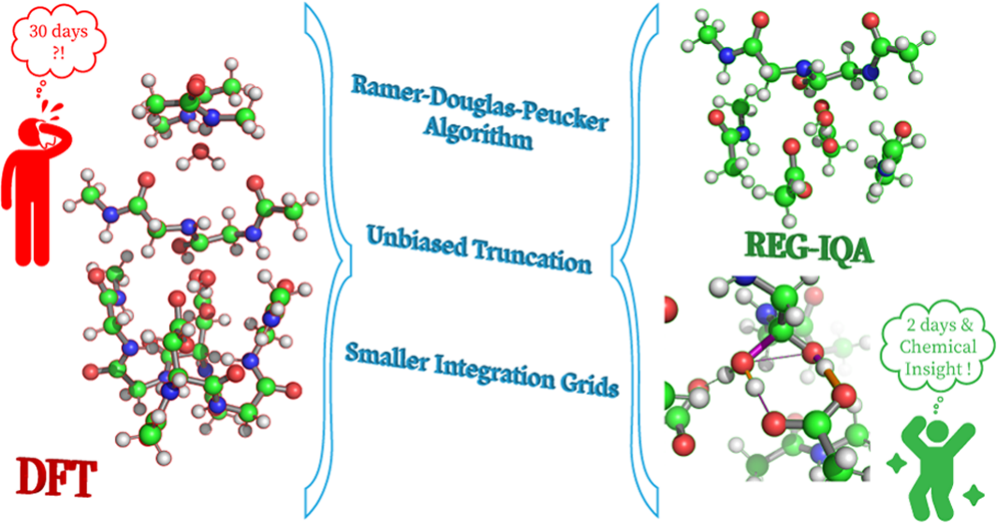

The relative energy gradient (REG) method is paired with the topological energy partitioning method interacting quantum atoms (IQA), as REG-IQA, to provide detailed and unbiased knowledge on the intra- and interatomic interactions. REG operates on a sequence of geometries representing a dynamical change of a system. Its recent application to peptide hydrolysis of the human immunodeficiency virus-1 (HIV-1) protease (PDB code: 4HVP) has demonstrated its full potential in recovering reaction mechanisms and through-space electrostatic and exchange–correlation effects, making it a compelling tool for analyzing enzymatic reactions. In this study, the computational efficiency of the REG-IQA method for the 133-atom HIV-1 protease quantum mechanical system is analyzed in every detail and substantially improved by means of three different approaches. The first approach of smaller integration grids for IQA integrations reduces the computational overhead by about a factor of 3. The second approach uses the line-simplification Ramer–Douglas–Peucker (RDP) algorithm, which outputs the minimal number of geometries necessary for the REG-IQA analysis for a predetermined root mean squared error (RMSE) tolerance. This cuts the computational time of the whole REG analysis by a factor of 2 if an RMSE of 0.5 kJ/mol is considered. The third approach consists of a “biased” or “unbiased” selection of a specific subset of atoms of the whole initial quantum mechanical model wave-function, which results in more than a 10-fold speed-up per geometry for the IQA calculation, without deterioration of the outcome of the REG-IQA analysis. Finally, to show the capability of these approaches, the findings gathered from the HIV-1 protease system are also applied to a different system named haloalcohol dehalogenase (HheC). In summary, this study takes the REG-IQA method to a computationally feasible and highly accurate level, making it viable for the analysis of a multitude of enzymatic systems.

## 1 Introduction

In the last few decades, computational techniques have become an integrated part of the drug discovery and drug design process thanks to their predictive capability. Indeed, these techniques currently constitute a crucial step in evaluating drug properties such as absorption, distribution, metabolism, elimination/excretion and toxicity (ADMET), kinetics, permeability, inhibition, and reaction mechanisms.^1−3^ A typical drug pipeline comprises five main stages: discovery, preclinical research, clinical trials, Food and Drug Administration (FDA) approval, and marketing. Computational and experimental techniques mostly take part in the first two stages.

From discovery to customer shelves, a typical drug development process can take as long as 20 years^4^ and has been estimated to cost an average of $2.6 billion.^5^ The early stages of this process involve discovering possible hit compounds, which can become lead compounds, eventually ready for optimization.^6,7^ This happens thanks to the use and recent development of computational techniques such as virtual screening (VS), molecular docking, and quantitative structure–activity relationships (QSAR).^8^ These are the cheapest and safest options when it comes to de novo drug design. However, within the computational community, it is well known that such techniques are less accurate than other methods but beneficial in terms of computational cost. The latter advantage is due to their dependence on large amounts of empirical data and the fact that they provide less atomic insight (i.e., less information) compared to quantum mechanics (QM). Ideally, if QM-based methods were as fast as those listed above, they would be used in the early stages of drug development given their accuracy.

Energy decomposition is a very important tool in the quest for chemical information extracted from quantum mechanical wave-functions because so many phenomena ultimately have their origin in energy, often at atomic level. Interacting quantum atoms (IQA)^9^ is a type of energy decomposition that does not suffer from the typical problems of more traditional decomposition methods, as critically reviewed^10^ alongside their variations, proposed in an attempt to overcome these problems. IQA is a reference-state-free and parameter-free energy partitioning scheme that is known for retrieving chemically intuitive energy terms from the wave-function. The IQA method is part of Quantum Chemical Topology,^11,12^ which is strongly related to QM. Indeed, “a topological atom is a quantum atom”.

The atomistic IQA energies (both intra-atomic and interatomic) can be fed to the so-called relative energy gradient (REG) method, which was proposed^13^ in 2018. Subsequently, REG computes chemical insight from these IQA energies. For example, REG can tell if a van der Waals complex is held together by hydrogen bonds, and if so, by which ones, and what their degree of covalency is. REG always needs a sequence of atomic energies, i.e., a dynamical change in the total system. Some control parameters such as the distance then governs this series of molecular geometries. In the case of this van der Waals complex, the change can be as simple as bringing together the two constituent monomers from infinity, as controlled by their intermolecular distance. In summary, and in more abstract terms, REG captures which individual atomic (i.e., local) IQA energies are primarily contributing to a relevant, dynamical change in the total energy of a system.

When combined into REG-IQA, the REG method has been used to explain a variety of phenomena such as the fluorine gauche effect,^14^ the biphenyl torsional energy barriers,^15^ the nature of complementary hydrogen-bonded networks in nucleobases,^16^ the directionality and nature of halogen bonds,^17,18^ and the nitrogen inversion of N-substituted aziridines,^19^ to name a few. These studies solve interesting questions by working with small systems. This “modus operandi” is well-known in QM calculations, where the results obtained for small representative systems are then transferable to larger, more realistic systems. However, in structural biology, which is at the root of the drug discovery pipeline, enzymes play a critical role. In this context, detailed mechanistic studies are prioritized in order to obtain accurate and reliable information about the interactions of a drug in a specific biological system.^20^ REG-IQA is capable of giving such detail at a high computational expense. Ideally, in an enzyme-catalyzed reaction, a full enzyme model of typically thousands of atoms should be employed but this is not feasible given the high computational cost. Methods such as the Quantum Chemical Cluster approach and QM/MM are well known for dealing with this situation at the best of their capabilities.^21−27^ However, one should keep in mind that IQA adds a layer of complexity to the already intricate QM calculations.

In 2018, the REG-IQA method was used for the first time on a system of considerable size (133 atoms)^28^ involving a protease. Other than intuitively explaining the already known mechanism of peptide hydrolysis in the human immunodeficiency virus-1 (HIV-1) protease active site, REG-IQA provided meaningful chemical insights into the interactions involved in this reaction. Exchange–correlation terms, which relate to the chemical concept of covalency (making and breaking of bonds),^29^ resulted in being the most relevant. It was rewarding that REG was able to rank, from more than 8000 interatomic exchange–correlation terms, only a dozen or so crucial interactions representing the concerted mechanism of hydrolysis. The study also revealed subtle catalytic effects related to so-called “through-space” interactions, which seems to be mysterious and confusing.

The ranking of the IQA energy terms that the REG analysis outputs is exhaustive and involves an enormous number of terms, i.e., 17,689 including exchange–correlation, electrostatic, and intra-atomic energies. The computational expense of IQA, as it was implemented at the time in 2018, is not really practical for a system of this size. The primary purpose of the REG-IQA method is “automating” the analysis of chemical systems, no matter how large, and to provide chemical insight while not having any prior knowledge of them. Indeed, out of *n*^2^ total possible interactions in a given system of *n* atoms, only a few will be highly relevant to the studied phenomenon. REG is able to capture these few. However, the computational demand of IQA is a bottleneck for this method, and there is a great need for a better strategy. Making routine use of REG-IQA is the goal of the current article.

In order to make REG-IQA an advantageous predicting tool for interactions in enzymatic active sites (or in any large QM system), two problems are explored as reported below.

- **Recovery Error**
  The recovery error is defined as the difference between the summed IQA partitioned energies and the total wave-function energy obtained from a QM calculation. Most previous studies (on much smaller systems) gave overall error smaller than 1.5 kJ/mol on the total energy of the systems obtained through IQA partitioning. However, the larger the system, the larger the error, given that there are more numerical integrations to be done. Indeed, for HIV-1 protease, the original paper showing 26 kJ/mol of recovery error, which should be contrasted with the Δ*E* of activation (around 66 kJ/mol), seems quite significant. However, as made clear later on, we are interested in energy gradients, which are not affected by a systematic error (energy shift). Second, the total energy of the system can well be of the order of −10 million kJ/mol such that the above recovery error amounts to only 0.0003%, and third, there may be error cancellations.
- **IQA Computational Expense**: Although IQA exhaustively analyzes every possible interaction in a system (both intra-atomic and interatomic) and without any bias as to where molecules start or stop (“atoms are atoms, are atoms”), the analysis is computationally expensive. The REG method relies on a dynamic change within a system, which is governed by control coordinate *s*. Hence, the number of intra-atomic and interatomic integrations that the program AIMAll^30^ has to calculate for a given system is
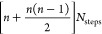
1where *n* is the number of atoms and *N*_steps_ is the number of points (i.e., single-point energies) of a chosen potential energy surface (PES). The first term of the sum is the number of intra-atomic integrations, while the second term is the number of pairwise atomic integrations. If both electrostatic and exchange interatomic interactions are considered, then this second number needs to be doubled, and the whole factor simply becomes *n*^2^.

Three main ways to overcome these issues constitute the main contribution of this article. The first consists of testing the limits of the IQA integrations and the resulting REG values through the tuning of quadrature grid parameters. The second way consists of systematically reducing the number of points on the PES analyzed with REG-IQA. The third way implies the analysis of a smaller, but representative, subset of atoms from which the same qualitative ranking can be extracted as that coming from the full system. Given the availability of previous calculations, the HIV-1 protease system is used as a benchmark for developing the current improved version of the REG-IQA method. The goal is to optimize the REG-IQA analysis while still obtaining relevant results. Finally, after benchmarking the HIV-1 protease, the methodology is directly applied to a haloalcohol dehalogenase (HheC) system, which has been recurringly studied in different density functional theory (DFT) benchmarks in the past decades.^31−33^

## 2 Methods

### 2.1 Interacting Quantum Atoms (IQA)

IQA is an energy decomposition scheme that was proposed by Blanco et al.^9^ It is based on the Quantum Theory of Atoms In Molecules (QTAIM)^34,35^ but generalizes its original definition of atomic energy by the virial theorem, which is only valid for stationary points. IQA provides chemical information by dividing the electronic wave-function into a few physically well-defined contributions from which all chemical phenomena can be explained. It does so by employing the first-order density matrix, ρ_1_, and the diagonal second-order density matrix, ρ_2_, as shown in eq 2
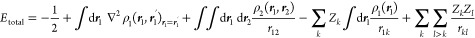
2where the first term represents the kinetic energy operator *T̂*, the second is the electron–electron potential *V̂*_ee_, the third is the electron–nucleus potential *V̂*_ne_, and the last one is the nucleus–nucleus potential *V*_nn_. The first three energies presented in eq 2 are obtained by solving the Schrödinger equation under the Born–Oppenheimer approximation. IQA benefits from the parameter-free, reference-state-free, and orbital-free nature of topological partitioning. Topological atoms are space-filling (i.e., no gaps, no overlap) and have unique three-dimensional (3D) volumes. An atomic basin Ω is defined by means of the zero-flux surface or interatomic surface (IAS) as
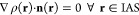
3where ∇ρ(**r**) is the gradient of the electron density and **n**(**r**) is the vector normal to the interatomic surface. Energy contributions are extracted by integrating over the topological volume *V*. Using Gauss’s divergence theorem, the 3D integral over an atomic basin can be transformed into a two-dimensional (2D) integral, as shown in eq 4
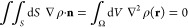
4where *V* is a variable of volume over which the atomic basin Ω is integrated, and ∇^2^ρ(**r**) is the Laplacian of the electron density. However, practically, eq 4 is obeyed only approximately, where a deviation from zero gives a measure of the numerical quality of the atomic integration. This small error affects the IQA energies, both intra- and interatomic, but the total effect can be monitored by the recovery error.

The total energy shown in eq 2 can be described in terms of simpler and chemically intuitive terms within the IQA framework as
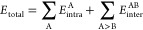
5where
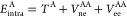
6
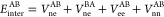
7Note that A and B are actually the atomic basins Ω_A_ and Ω_B_. Equation 6 shows the partitioning of the so-called intra-atomic energy (i.e., energy within the atom), which consists of kinetic energy (*T*_A_), nucleus–electrons attraction (*V*_ne_^AA^), and electron–electron repulsion (*V*_ee_^AA^) energy. Equation 7 is the total interaction energy between two atoms A and B and is composed of nucleus–nucleus repulsion (*V*_nn_^AB^), nucleus–electron attraction (*V*_ne_^AB^ and *V*_ne_^BA^), and electron–electron repulsion (*V*_ee_^AB^). The latter can be further split into classical electron–electron interaction (*V*_ee,cl_^AB^) and exchange–correlation (*V*_xc_^AB^), making the definition of interatomic energy as
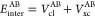
8In principle, *V*_xc_^AB^ can also be divided into exchange *V*_x_^AB^ and correlation *V*_c_^AB^. In practice, this is not feasible for larger-cluster models due to the very high computation times for calculating electron–electron correlation. Moreover, enzymes are typically modeled through the more computationally efficient Kohn–Sham density functional theory (KS-DFT) with hybrid and nonhybrid exchange–correlation functionals. Despite the lack of the second-order density matrix in its approximation, DFT has been made compatible with the IQA formalism for the B3LYP functional^36^ and later generalized for more exchange–correlation functionals.^37^ The peculiarity of energy decomposition schemes such as IQA is that the partitioned energy terms are strictly related to chemical intuition. Within IQA, the following associations can be made:

- *V*_xc_^AB^ = covalency, bond order, and hyperconjugation.^29,38^
- *V*_cl_^AB^ = charge transfer, ionicity, and polarity.^14,28,39,40^
- *E*_def_^A^ = steric effects.^41−43^

The energy terms described in this section can be fed to the new inhouse program^44^ REG.py, which performs the REG analysis.^45^

### 2.2 Relative Energy Gradient (REG)

The REG method offers the capacity to determine the most relevant interactions along any PES. The technique has been successfully applied with IQA energies but it is not restricted to them. It is a broad method that can also be used with other energy partitioning schemes, which is why its general form is presented here. REG solves two main issues encountered when studying energetically partitioned systems: (i) detecting the interactions that contribute most to the PES, and (ii) extracting chemical insight. The latter mostly depends on the chosen energy decomposition scheme, which in the IQA case is parameter-less and reference-state-free.

Note that REG analysis is commonly performed on segments of a PES. Specifically, a system evolves dynamically as governed by a control coordinate *s*. The latter generates a set of geometries obtained through an intrinsic reaction coordinate (IRC) or a PES scan (i.e., bond distance, angle, or torsion). The energy curve is then divided into segments by searching for stationary points. Indeed, a segment is defined as a part of the PES between two subsequent stationary points. These can be energy minima, maxima, or saddle points, for example reactants, products, or transition states. The reason this separation is pursued is mainly because a single strongest interaction cannot adequately represent a whole PES. Indeed, as already pointed out in the original REG paper for the Lennard-Jones 12-6 potential,^45^ standard chemical intuition separates the PES into two parts connected by an energy minimum. Each part is described, respectively, by a specific potential: repulsive or attractive. More details on this are presented in Section S1.3.

IQA is an additive energy decomposition method, which means that the sum of all of the computed single energy terms strives to be equal to the “true” wave-function energy (*E*_tot_)
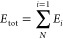
9where *E*_*i*_ is the *i*-th partitioned energy term and *N* the total number of partitioned energy terms. The IQA terms present are *E*_intra_, *V*_cl_, and *V*_xc_, which leads to a total number of terms equal to *n*^2^ [=*n* + *n(n* – 1)/2 + *n*(*n* – 1)/2], with *n* being the number of atoms. The word “strives” used earlier hints at the fact that eq 9 is not quite an exact equality. For example, within IQA, there is an intrinsic mathematical error to each energy term caused by the numerical integration over atomic volumes. However, we will demonstrate later that when an integration over the volume of the topological atoms is computed correctly then this mathematical error does not affect the qualitative outcome of a REG-IQA analysis.

The REG method takes every energy term and generates a linear relationship with the total energy *E*_tot_ at each point of the control coordinate *s* as shown in eq 10
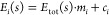
10

where *m*_*i*_ is the linear coefficient and *c*_*i*_ the intercept. These linear relationships are fitted over *M* data points to extract valuable chemical information during the dynamical evolution of the system. Hence, a REG value for the *i*-th energy term along *s* is obtained by means of an ordinary least-squares regression as shown in eq 11, where τ marks the transpose
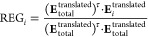
11where
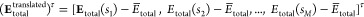

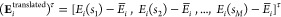
Both total energy and partitioned energies are translated over their respective means. This is done to make energy profiles comparable, and it is a critical step in the calculation of the relative energy gradient. Note that the REG value depends only on the energy gradients of the system but not on the actual values of the control coordinate. Thus, this method can be applied to any movement regardless of whether it is in space or time. Given that linear regression is used to explain the relationship between a partitioned energy term and an actual energy profile, an error metric is necessary to assess the viability of this approach. This job is carried out by the Pearson correlation coefficient (*R*_*i*_), as defined in eq 12
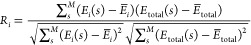
12A pair of values is defined for each energy term of a specific system: a REG coefficient and a Pearson coefficient. Terms for which *R* is low are discarded; they express poor linear correlation, which means that the REG relationship breaks down. However, for respectable values of the Pearson coefficients, REG coefficients are then ranked from most positive to most negative for each segment of a PES. Positive terms means that the corresponding energy contributions work with the curve trend, which means that they have the same gradient as that of the total energy in that given segment. On the other hand, negative terms work against the trend of the total energy; in other words, they have an opposite gradient to that of the total energy. The higher the absolute REG value of a given (atomic) energy contribution, the more it “calls the shots” along that energy segment, and thus characterizes and explains the total energy profile.

### 2.3 Computational Details

Details of the HIV-1 protease 133-atom system and IRC scan can be found in the original paper,^28^ and details of the HheC system are in their own original references. In practice, only single-point energy calculations were carried out in this study given the availability of the previous 11 points (i.e., geometries with single-point energies) of the IRC. All of the geometries were obtained at the B3LYP/6-31+G(d,p) level of theory. The initial transition-state structure for the HheC system was obtained from the literature and reoptimized at the M06-2X/Def2TZVP level of theory with the D3^46^ dispersion correction and zero damping. A vibrational frequency analysis was performed on the obtained transition state, and geometries along the most intense imaginary frequency were generated. These geometries correspond to the expected reaction coordinate for the HheC reaction. All DFT calculations were performed using the program^47^ GAUSSIAN16. The QTAIM and IQA calculations were performed with the program^30^ AIMAll 19.10.12 using both default and custom integration parameters. Specifically, a maximum of eight CPU cores on heterogeneous hardware were used for each point on the IRC (with hyperthreading turned off). Note that using default integration parameters with AIMAll 19.10.12 means that the program will start the atomic basin integrations with a fairly low number of grid points (≤4500) for the outer angular quadrature and will automatically increase this number in case the integration error (as expressed in eq 4) for an atom is poor.

REG analysis was carried out with the updated inhouse code package^44^ REG.py, which supersedes the program ANANKE. Visualization and model handling were executed with the program^48^ GaussView6, the open source package^49^ PyMol, and a custom^50^ PyMol-REG plugin.

## 3 Results and Discussion

The following results compare interatomic (*V*_xc_, *V*_cl_) REG-IQA values obtained for the 133-atom system that is the HIV-1 protease. There will be no focus on the chemical description of the obtained REG-IQA terms because it is not that relevant in the current context of algorithm improvement. However, we work with a real system and the details of its reaction mechanism can be found in the original paper.^28^ Results will be described as a thought process, from ideas to concrete algorithms and workflow. Details on computational efficiency will also be included. Finally, the outcomes learnt from the HIV-1 system will be directly applied to the 112-atom HheC system.^33^

### 3.1 REG Stationary Points Dependency

As an introduction to the discussion, we mention some initial thoughts on the REG-IQA results’ dependency on the number and positions of points (i.e., geometries) along the PES of the HIV-1 protease system.

**Figure 1:**
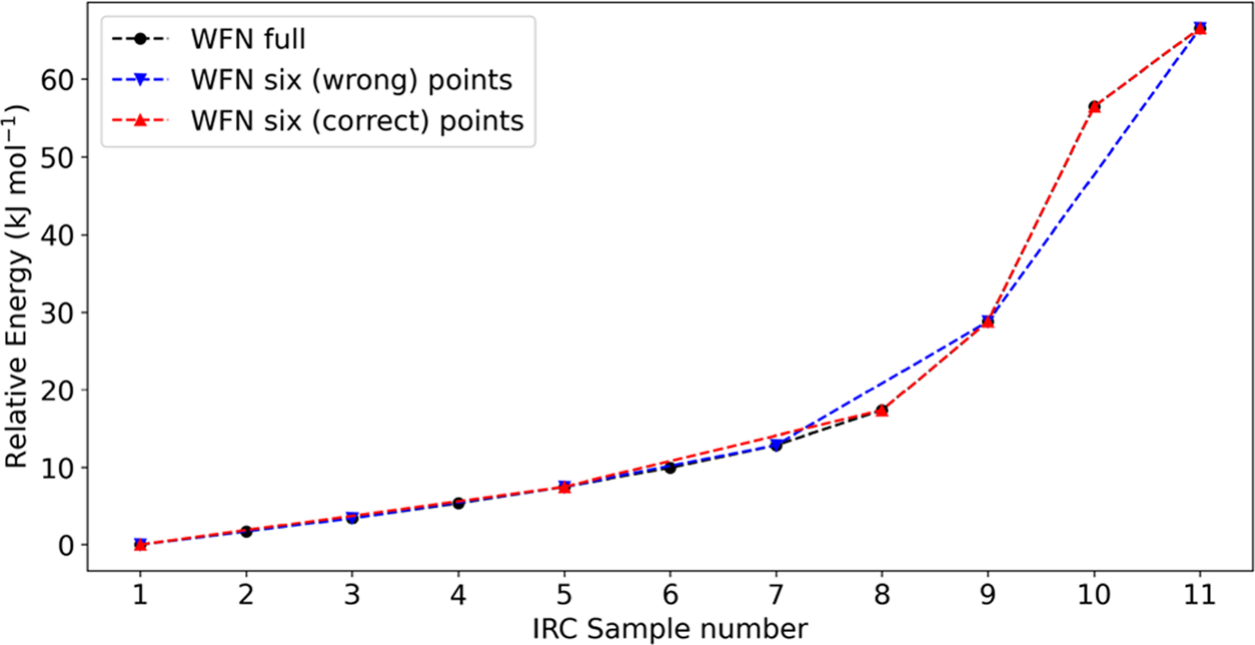
Energy profile of the reaction for complete HIV-1 protease hydrolysis for different selections of geometries. The original 11 selected geometries are marked in black, the (wrong) selection of six points in blue and in red the (correct) selection of six points.

The reaction mechanism depicted in Figure 1 is the reference point for the following study. This shows the specific segment of the PES that is analyzed throughout the manuscript. More specifically, the segment corresponds to the energy of activation (from reactant to transition state) of the peptide hydrolysis of the substrate mediated by HIV-1 protease. As mentioned in the Section 1, the first step to speed-up the REG-IQA analysis is choosing the best possible stationary points along the PES or IRC.

**Figure 2:**
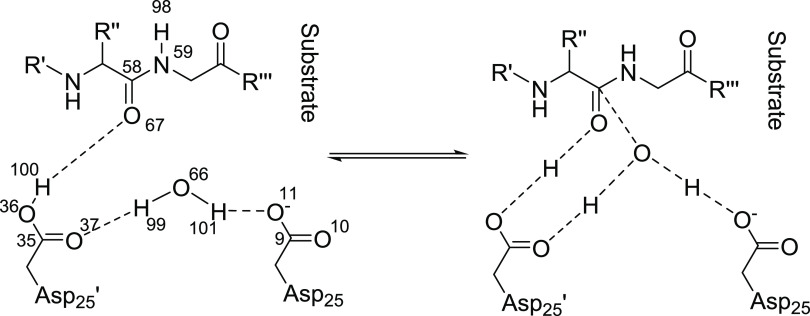
Scheme of the HIV-1 protease peptide hydrolysis reaction near the active site. Reactants are on the left, while the transition state is on the right of the central arrows.

Figure 2 represents the IRC for the HIV-1 protease hydrolysis. The original DFT calculations were pursued on more than a hundred geometries but only 11 arbitrarily chosen geometries were used in the following REG-IQA analysis. The principle that should be followed for selecting the correct set of points is keeping the shape of the PES as authentic as possible, i.e., similar to the original QM calculation. Indeed, the REG-IQA method acts on energy gradients between stationary points. Thus, the choice of the points along the PES strongly affects both the reliability and computational efficiency of REG-IQA. Here, we will give an arbitrary approach to the HIV case study in order to showcase how the choice of stationary points affects the reliability of the REG analysis. Moreover, we will provide a formal and rigorous way to select a certain number of geometries along a PES in Section 3.2.

**Figure 3:**
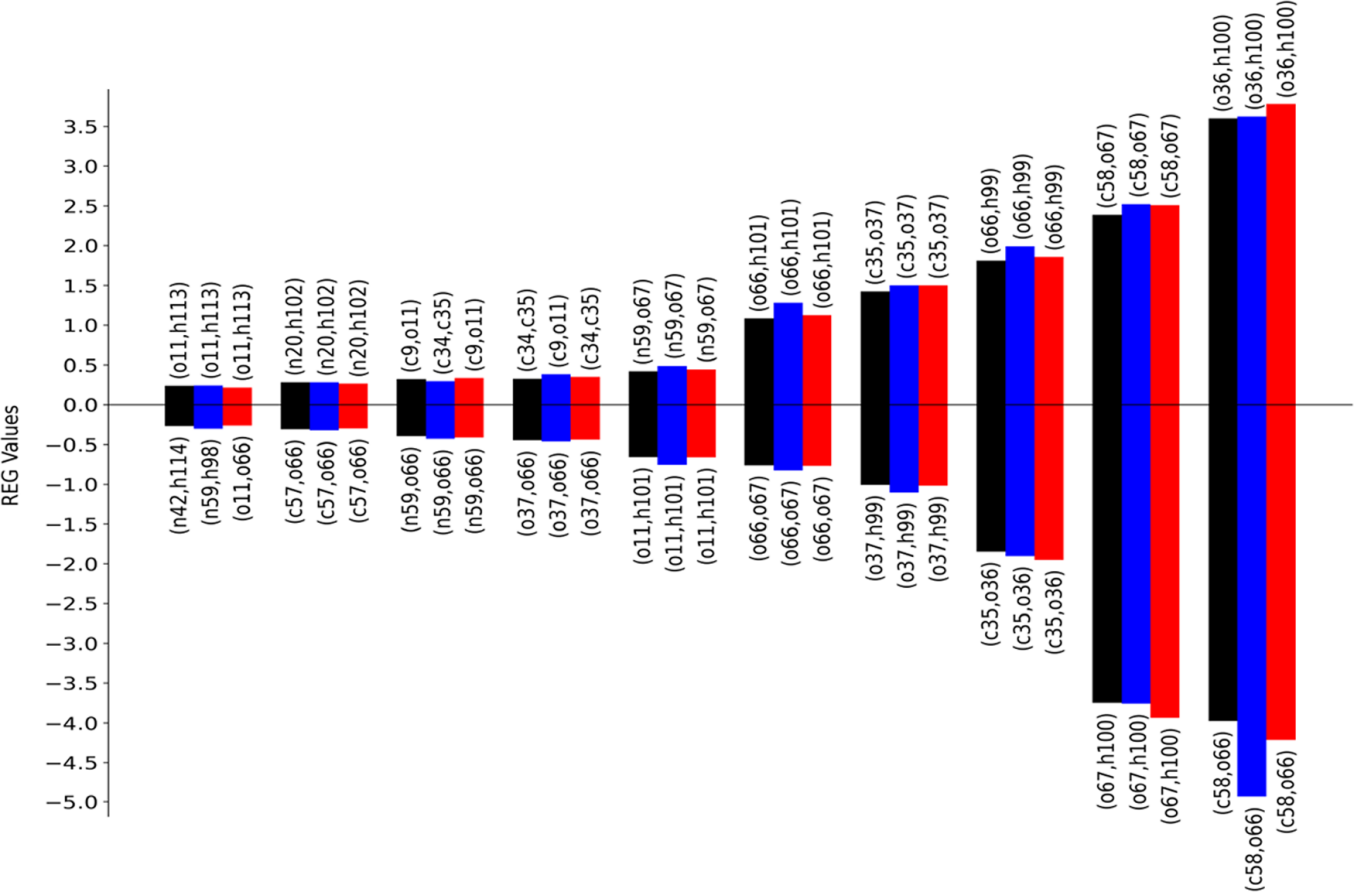
REG-*V*_xc_ values for the analyzed PES. Only the 20 (10 positive and 10 negative) most relevant interactions are shown. Coloring corresponds to that of Figure 1. Each value is labeled with the corresponding atom pair. Pearson correlation coefficients are shown in Tables S1–S3.

**Figure 4:**
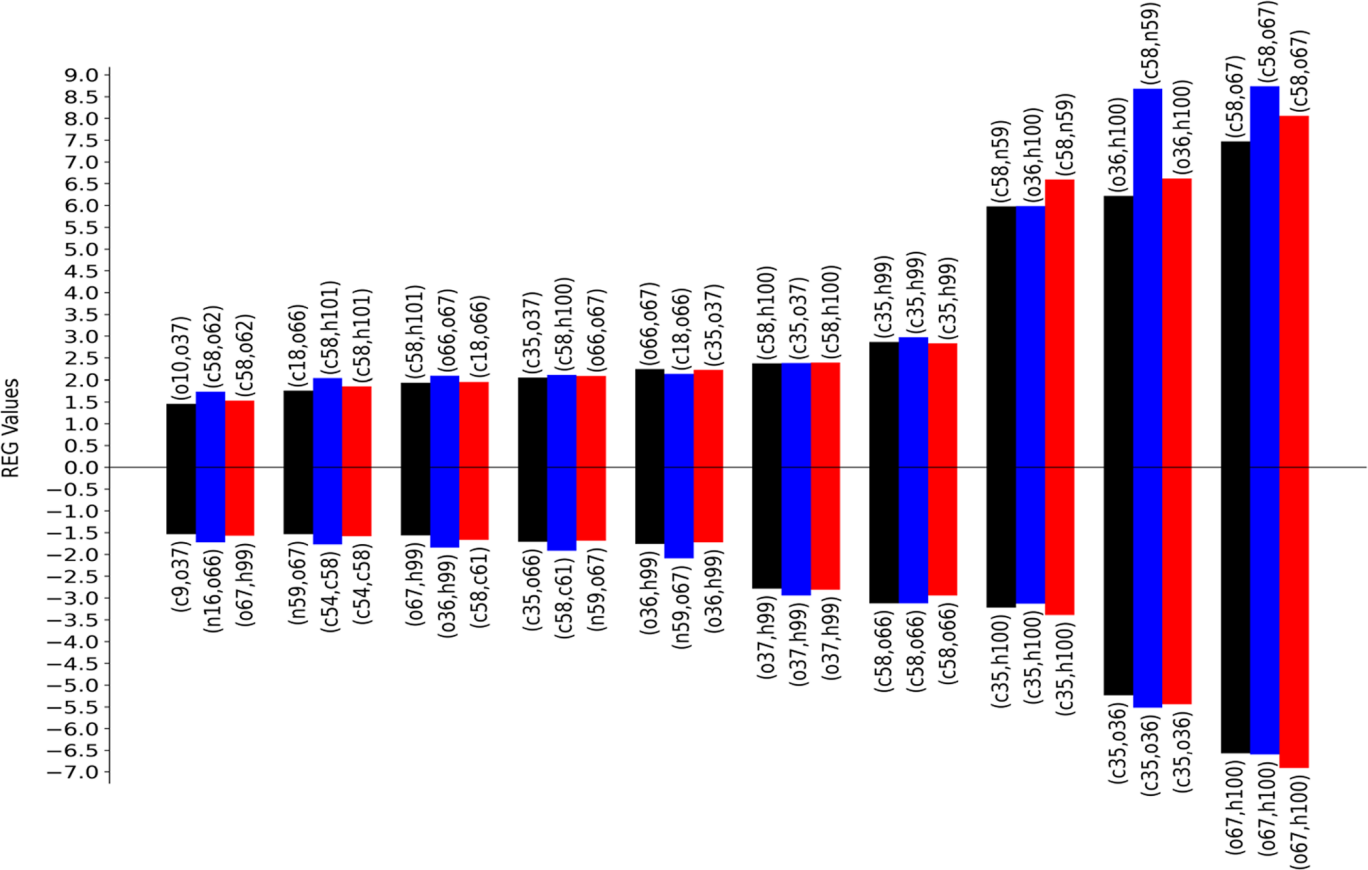
REG-*V*_cl_ values for the analyzed PES. Coloring corresponds to Figure 1. Only the 20 most relevant interactions are shown. Each value is labeled with the corresponding atom pair. Pearson correlation coefficients are shown in Tables S1–S3.

Figure 1 shows the first example of decreasing the number of points from the original number of 11 (black). The blue curve encompasses points 1, 3, 5, 7, 9, and 11 of the original PES, while the red curve contains a different set of six points: 1, 5, 8, 9, 10, and 11. The REG values for each case (red, blue, black) are shown in Figure 3 (*V*_xc_) and in Figure 4 (*V*_cl_). Which atom pairs the various colored bins refer to is not important; indeed, the labeling is just for the sake of completeness. The purpose is to find out if the relative importance and thus the ranking of the REG values changes between a subset of points and that of the full original set. We see that the ranking of REG values for the blue curve is very similar to that of the original 11-point PES but two minor issues should be pointed out. One issue is that the REG ranking mainly starts to differ after the ninth term on both the positive and negative sides. Indeed, considering 6 points in the PES instead of 11 changes its shape and thus the REG coefficients. However, not all interactions shown in these tables are relevant for the reaction. The second issue is seen for the electrostatic terms *V*_cl_(o36,h100) and *V*_cl_(c58,n59), which change their ranking (and have higher values) compared to the reference calculations. One possible reason is that the PES is not well represented with these six REG geometries. Indeed, a comparison with the original (“true”) PES in black shows a different curvature in the interval marked by 9, 10, and 11. This suggests that taking into account point 10, where there is an inflection in the curve, should better represent the PES. In light of the two issues mentioned above, a REG analysis is presented for the six points, labeled 1, 5, 8, 9, 10, and 11, which constitute the red curve. A comparison between the results of the blue curve (6 points) with those of the black curve (11 points) demonstrates how both REG ranking and values are reestablished due to the better representation of the original 11-step PES curve, which is the reference point of the whole analysis. Overall, using fewer steps on the PES is feasible given that the qualitative narrative of the REG analysis has not changed and the REG values shift by a negligible amount. More importantly, the computational demand is almost halved in going from 27.8 × 11 = 306 CPU days down to 27.8 × 6 = 167 days, on eight CPU cores for each geometry (see Section S6).

### 3.2 IQA Grids Benchmark

As stated in the Methods section, IQA integrations are numerical, and their accuracy depends on the mesh and number of grid points chosen for every atom. Here, we perform a preliminary benchmark where default settings of the program AIMAll are compared to the lowest (i.e., cheapest computationally) possible integration settings in order to investigate if a qualitative (or even quantitative) REG-IQA analysis can be obtained at lower computational cost.

For default settings (i.e., “mesh = fine” and “outer angular quadrature = automatic”), employing eight CPU cores for each point on the total system’s PES demands the excessive total CPU computation time of 27–29 days. Calculations were more than three times faster than older versions of AIMAll, which demonstrates the better parallelization and overall stability of the newer version of AIMAll. Turning to the cheapest possible custom settings (“mesh = sparse” and “outer angular quadrature = GS1”), the calculations took an average of 10.3 days, resulting in an additional 3-fold speed-up. Section S2 compares the REG-IQA analyses for default and custom settings. In summary, the chemical information gleaned from REG-IQA is preserved at a much lower computational cost using smaller (“lower”) grids.

Nevertheless, the choice of this lower setting worked well for the HIV-1 protease but may not do so for other systems because the accuracy of the integrations is system-dependent. Thus, we recommend to still employ default AIMAll settings because if the numerical accuracy for the starting grid (GS4 ≤ 4500 points) on a given atom is low, then AIMAll will automatically perform a new integration for that atom with better grid settings. Here, we demonstrated for the HIV-1 protease that, if lower amount of resources are available, then the lowest integration settings can dramatically decrease the computational expense at no loss in qualitative and quantitative chemical information.

### 3.3 Ramer–Douglas–Peucker (RDP) Algorithm

The first part of a REG-IQA analysis is the selection of the number of geometries that define the ab initio PES. Section 3.1 demonstrated that considering a poor subset of points can lead to an unreliable REG study, while choosing too many points considerably increases the computation time. Here, we present a simple point-detection algorithm that systematically selects the least number of points in a PES and introduces only one parameter to achieve this. This approach works on any given curve, and thus it is assumed that the PES is correctly calculated prior to the usage of this algorithm.

The Ramer–Douglas–Peucker (RDP) algorithm is a line-simplification algorithm primarily used in image processing and cartography^51−54^ using the concept of a polyline or segmented curve. This algorithm was initially developed for processing vector graphics, reducing memory usage of an image, smoothing graphics, or even minimizing noise. Its simplicity led to many more upgraded versions in different areas. In this study, we employ the RDP algorithm to obtain a minimal number of points to define a PES while preserving its overall shape. This culling of points will in turn reduce the computational cost of the whole REG-IQA analysis.

**Figure 5:**
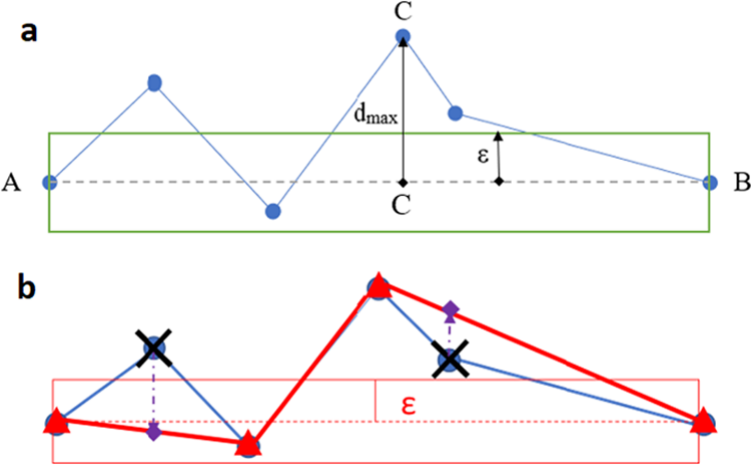
(a) First step of the Ramer–Douglas–Peucker algorithm for a polyline (in blue). *d*_max_ is the maximum perpendicular distance from the polyline defined by points A and B, here shown as CC′. If this distance is larger than ε, then point C will be kept. It means that this point is essential for keeping the overall shape of the polyline, at least according to user-defined threshold ε. Had ε been much larger, then C would have been eliminated. After this action, the algorithm continues with the interval AC and CB. The algorithm carries on recursively until all subintervals have been checked for possible point elimination. (b) Finalized Ramer–Douglas–Peucker algorithm. The polyline defined by red triangles and red segments is the final polyline after the RDP with tolerance ε applied to the original blue polyline. The eliminated points (black crosses) are then projected in one single direction (purple arrows) onto the final polyline. This linear interpolation makes possible the root mean squared error (RMSE) comparison of the original blue polyline, and the newest minimized polyline enhanced with the interpolated points (purple diamonds).

Figure 5a helps understanding the algorithm by means of an example. First, we introduce the parameter ε > 0, which acts as a user-defined threshold or tolerance. We start with the extremities of a given polyline: the two points A and B. The algorithm then finds the point on the polyline that is the farthest from the line segment that is bound by the first and last points (dashed line in Figure 5a). This farthest point is point C because the “perpendicular distance” between any point on the polyline and the dashed line reaches a maximum at this point, marked by CC′ or *d*_max_. If *d*_max_ is larger than ε, then that point (C) is kept, or otherwise it is discarded. If a point is kept, then the polyline is split into two new segments, and the algorithm proceeds recursively until all of the perpendicular distances have been checked against ε.

Another helpful approach, called the Visvalingam–Whyatt algorithm, accomplishes a similar purpose but with a different principle: the tolerance (or ε) in this case is the area of the triangles defined between three points of the polyline.^55^ However, RDP is faster and is better at minimal simplification,^56^ which follows the point of view for the REG-IQA minimization presented in this work. The main issue that comes with the RDP algorithm is its parametric nature. In the context of PESs, the tolerance value does not have an actual physical (or chemical) meaning and is thus challenging to choose. A nonparametric way has been explored by Prasad et al.^57^ Still, it mainly works in the context of digitization and images, which are based on integer numbers (pixels) and not floating point numbers as for PESs. Therefore, a simpler way to overcome the problem of parameterization is discussed next along with how to convert the ε value to an energy, as a root mean squared error (RMSE).

PESs can be seen as two-dimensional (2D) images. Indeed, a PES is defined in Cartesian coordinates (*x*,*y*) where the abscissa is the displacement and the ordinate is the potential energy. Hence, it is possible to apply the RDP algorithm. Note that it is also possible to apply this algorithm to 3D images^58^ although this is not necessary in the context of REG-IQA given its one-dimensional (1D) displacement (the control coordinate *s*). A straightforward way to make RDP useful in this scenario is to convert the parameter ε to a well-known physical quantity: potential energy. We do this by applying the RDP algorithm on a chosen PES from the minimum value of ε (which is equal to zero) to the maximum possible deviation with a chosen step size and obtain a new polyline for each tolerance. The maximum possible value of ε is simply found using the RDP algorithm on the first and last point of the chosen polyline. The step size for the ε scan is arbitrary but, given that RDP is simple and very efficient, a small value of 0.01 was sufficient for the PESs employed in this study. Lower step sizes could be necessary depending on the shape of the analyzed PES. Moreover, this approach avoids the possibility of retrieving a new polyline as a single line between its first and last point, which would not be chemically meaningful as it would not represent the sigmoidal shape of a typical PES (e.g., from reactant to transition state). A quick useful discussion on the units of the original tolerance ε is presented in Section S4.

Once each new polyline (i.e., a minimized PES) is obtained, an interpolation with the original PES creates a set of new points that can be assessed with the RMSE metric (Figure 5b). The RMSE is obtained by comparison of the original PES and the newly interpolated polyline. This new measure, which does not appear in the original RDP algorithm, allows one to essentially monitor the effect of the new polyline, generated by a fully converged recursive RDP with a given tolerance ε, by means of an energy difference. Given an RMSE threshold value, the algorithm will output a polyline with fewer points for that specific value. Thus, a more accurate (i.e., lower RMSE) minimized PES will have more points, while a less precise (i.e., higher RMSE) minimized PES will have fewer points. The advantage is that the RMSE is in energy units and is independent of the control coordinate. Thus, one can quantitatively obtain a minimized PES depending on the desired accuracy, which will then translate into the reliability of the REG-IQA analysis.

Note that RDP works for any given initial polyline. This means that RDP does not discriminate between few points or many points, nor does it care about how the curve is shaped. However, given the nature of REG-IQA, it is most suitable to run RDP on each segment of the PES rather than on the whole PES. This is because the risk of eliminating critical points (i.e., maxima and minima) in the PES should strictly be avoided. Moreover, in a well-defined PES containing many points, the algorithm will work effortlessly given its negligible computation times. As previously mentioned, this approach assumes that a correct PES is employed for the complete REG-IQA analysis; hence, no poorly defined original PES should ever be considered.

**Figure 6:**
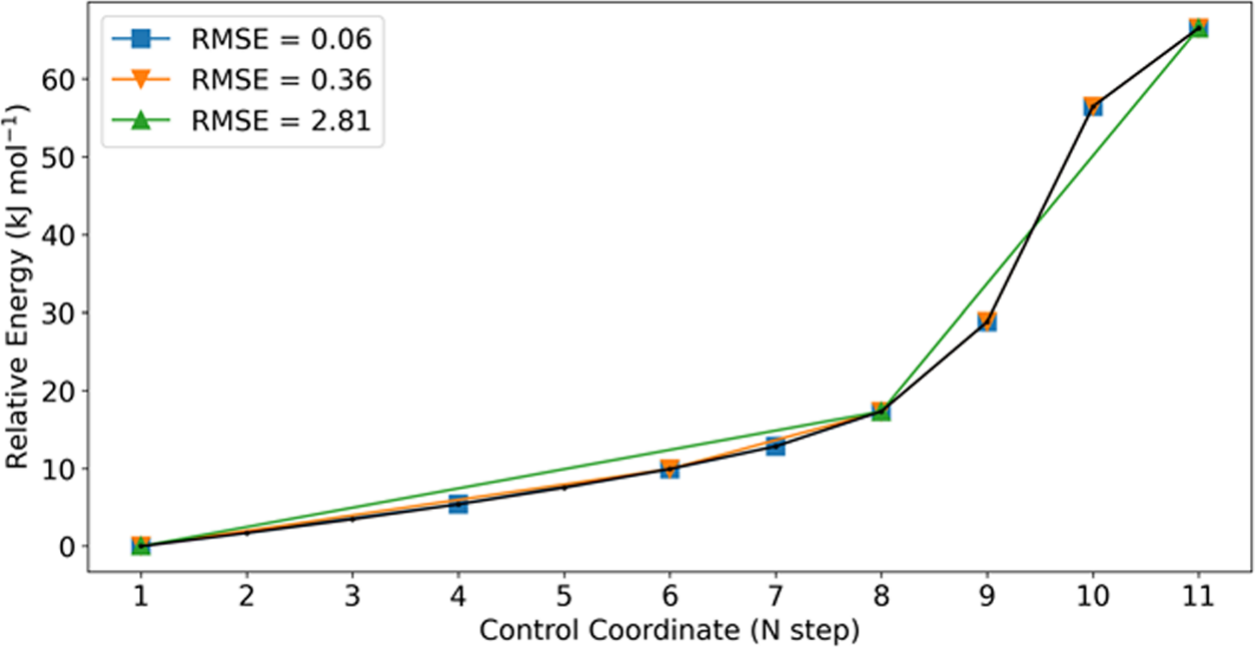
HIV-1 protease hydrolysis PES with chosen RMSE tolerance of (blue) 0.1, (orange) 0.5, and (green) 3.0 kJ/mol. The relative RMSE (kJ/mol) for each minimized curve is shown. The black polyline represents the original PES (11 points) as in Figure 1.

Applying the RDP algorithm to the HIV-1 protease case study, Figure 6 shows that the polyline obtained with an RMSE = 0.36 kJ/mol is almost equal to the arbitrary approach of Section 3.1 where six geometries of the PES were manually chosen for the REG-IQA analysis aside from point 6. However, this time the selection is made with a more elegant and consistent method. More examples on the RDP applied to PES are shown in Figures S2 and S3.

### 3.4 Selection of a Subset of Atoms

We now present two different ways of approaching the selection of a subset of atoms to lower the computational time of the IQA analysis with corresponding results. The first way involves the biased selection of atoms belonging to the complete system wave-function, while the second way consists of employing a smaller wave-function using an unbiased approach. Throughout this section, the subset of selected atoms will be referred to as the “lite” system, while the 133-atom system will be referred to as the “full” system.

#### 3.4.1 Biased Selection Using the Full Wave-Function

The general protocol proposed for this approach is as below:

- Model the full system and obtain a PES, such as the scan of an internal coordinate or an intrinsic reaction coordinate.
- Run the RDP algorithm with a chosen RMSE and find the smallest number of initial geometries that best represent the PES.
- Obtain the full-system wave-function for each geometry.
- Arbitrarily select the chemically more relevant atoms of interest to the study, defining the lite system.
- Calculate the IQA energies for the lite system.
- Run a REG analysis only on the lite system.

This protocol makes the REG analysis more qualitative than quantitative. Indeed, the recovery error (RMSE between wave-function energies and the sum of IQA energies) is not a valid quantity anymore due to the entirely different energies of the full system and the subset of atoms chosen. Previously, it was pointed out that REG acts on energy gradients (i.e., energy variations) rather than absolute energies. Hence, the chosen subset of atoms should be “good enough” to represent a PES as close as possible to the PES of the full system. The way that atoms are chosen in this approach depends purely on chemical intuition. In HIV-1 protease peptide hydrolysis, most of the interactions are likely close to the enzyme pocket and to the reaction center, i.e., where the reaction happens. In other cases, such as enzyme inhibition, many interactions occur between the surface of the enzyme pocket and the substrate. Long-range interactions also occur but are generally less relevant than those at a close range.

**Figure 7:**
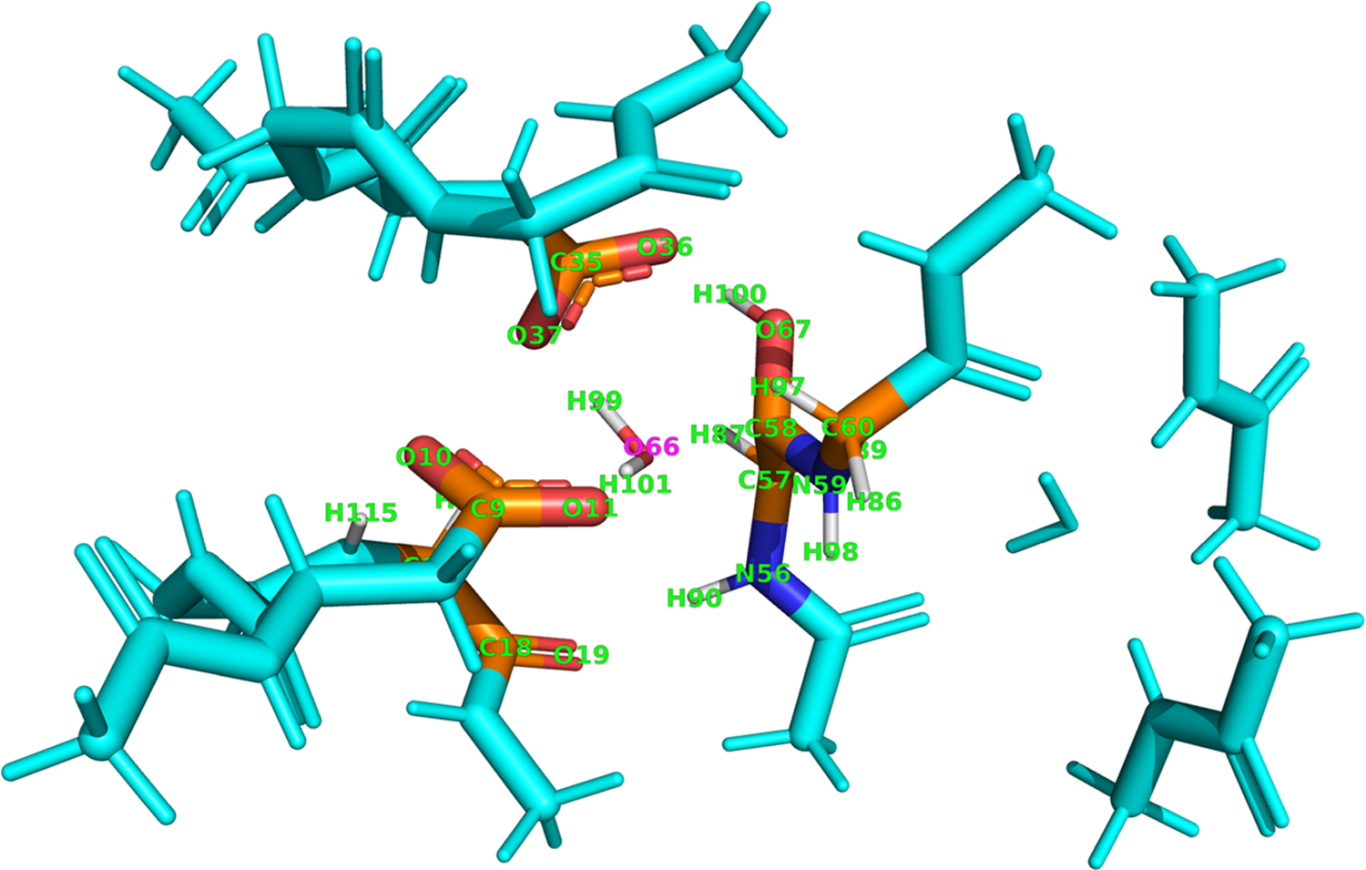
HIV-1 protease-active site 133-atom model. Lite system in orange with green and magenta labels and the rest in cyan. The lite system consists of 27 atoms (all of which are labeled, and only they) on which the REG-IQA analysis was performed.

In the current HIV-1 protease benchmark, unlike in the procedure in the paper by Thacker et al.,^28^ the atoms are picked by creating a sphere of chosen radius *r*, centered at an arbitrary point in space, which can be the center of an enzyme-active site for example. The reference point is atom o66 (magenta label in Figure 7) of the water molecule involved in the reaction and *r* = 4.0 Å (Figure 7). Also, the stationary point (1) was chosen as the geometry of reference. Note that different means of choosing the subset of atoms can be explored.

Calculating IQA energies for 27 atoms in the 133-atom wave-function guarantees an overall faster protocol and a lower computational expense, given the much lower number of integrations compared to an all-atom calculation. Indeed, the computation time for one single geometry of the IRC takes ∼3 days on eight CPU cores. Comparing Table S3 with Table 1 shows how REG values, REG rankings, and Pearson correlation coefficients are identical up to the listed precision. This happens because the full-system wave-function is employed in these calculations. Hence, there is no loss of information in the REG results even if only a subset of 27 atoms is used for the IQA analysis. Choosing these atoms means that the computed energetic contributions (specifically,  = 729) are the most relevant for the reaction, as expected.

**Table 1:** HIV Protease Full-System *V*_xc_ (Left) and *V*_cl_ (Right) REG Values on Points 1, 5, 8, 9, 10, and 11 of the Original PES

term	REG	*R*		term	REG	R
*V*_xc_(c58,o66)	–4.2	–0.89		*V*_cl_(o67,h100)	–6.9	–0.96
*V*_xc_(o67,h100)	–3.9	–0.96		*V*_cl_(c35,o36)	–5.4	–0.97
*V*_xc_(c35,o36)	–2.0	–0.96		*V*_cl_(c35,h100)	–3.4	–0.96
*V*_xc_(o37,h99)	–1.0	–0.98		*V*_cl_(c58,o66)	–2.9	–0.95
*V*_xc_(o66,o67)	–0.8	–0.98		*V*_cl_(o37,h99)	–2.8	–0.99
						
*V*_xc_(o66,h101)	1.1	0.93		*V*_cl_(c58,h100)	2.4	0.98
*V*_xc_(c35,o37)	1.5	0.96		*V*_cl_(c35,h99)	2.8	1.00
*V*_xc_(o66,h99)	1.9	0.97		*V*_cl_(c58,n59)	6.6	0.76
*V*_xc_(c58,o67)	2.5	0.96		*V*_cl_(o36,h100)	6.6	0.94
*V*_xc_(o36,h100)	3.8	0.96		*V*_cl_(c58,o67)	8.1	0.90

Moreover, the specific energy values obtained from the integrations on the lite system are equal to the energy values obtained for the full system. As an example, the (o58,o67) IQA interatomic energies (in au) are presented in Table S5. Although the absolute energy obtained from summing up the IQA terms is on average −900 au, which is almost 2600 au more than the energy obtained for the full-system wave-function energy, the REG method is not dependent on the total sum of IQA energies. Instead, REG acts on the original ab initio energy and the specific IQA interaction energy fed to it. The only dependency REG has within the biased approach is the choice of the subset of atoms: the more relevant the subset, the more reliable is the REG analysis outcome. Note that Table 1 only shows 10 highest REG values out of 729 possible values. It is straightforward to understand that the REG ranking starts to diverge at lower values from the 133-atom system results given the unaccounted remaining 106 (=133 – 27) atom interactions. However, it is arguable that the interactions involving the latter are not as relevant as those from the selected 27 atoms for the peptide hydrolysis.

**Figure 8:**
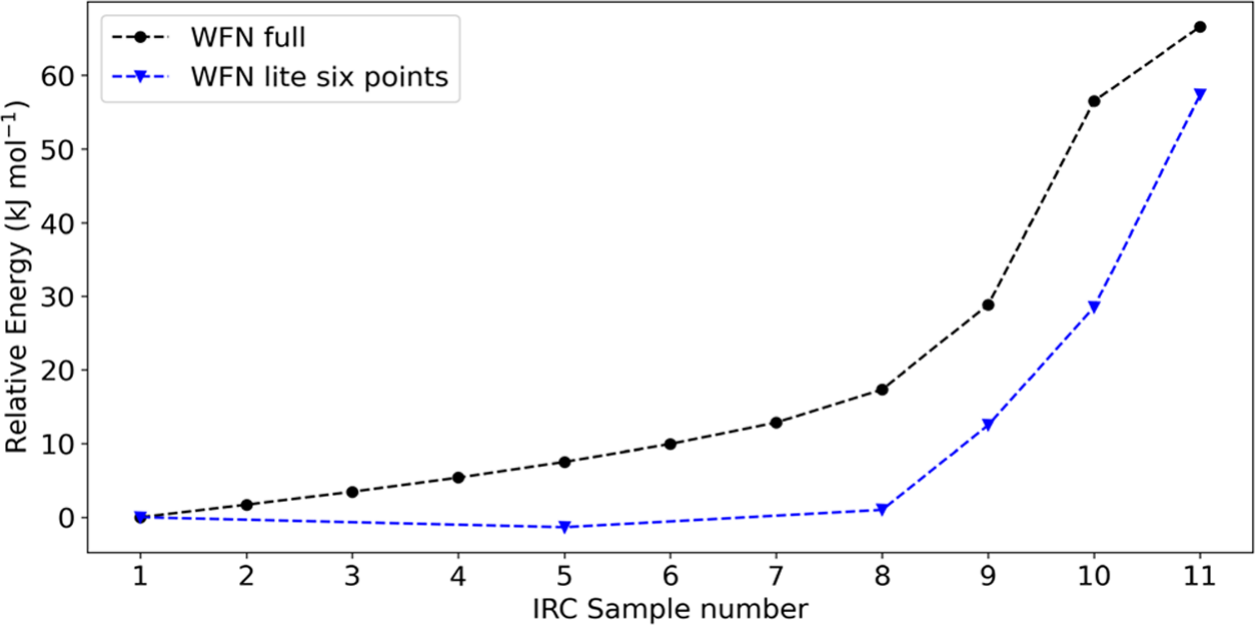
Intrinsic reaction coordinate from reactants to the transition state for the lite system (27 atoms) in blue and the original 11-point 133-atom system in black.

**Figure 9:**
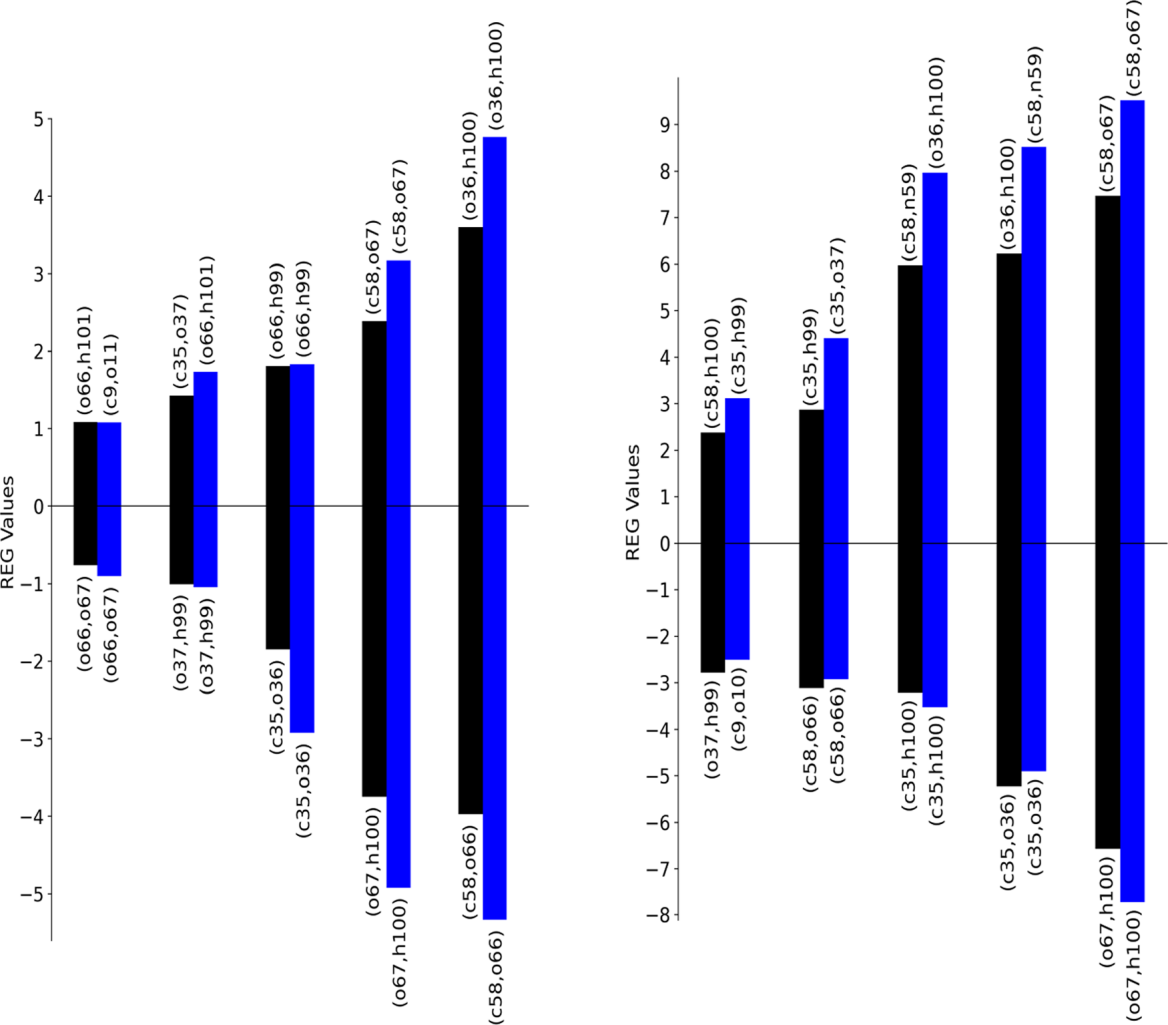
REG-*V*_xc_ (left) and REG-*V*_cl_ (right) values for the PESs shown in Figure 8. The REG values of the lite system (27 atoms) are in blue and those of the original 11-point 133-atom system are in black.

One question that may arise from this approach is this: if 27 atoms are enough to represent the reaction mechanism, then why was the 133-atom system used in the first place? The quick answer is that a larger system is necessary for a more accurate description of the whole enzyme behavior, which a smaller system most likely would not retrieve. In particular, there are two ways the system is treated. First, optimized geometries with QM calculations (DFT in this case) are obtained on a larger system. This must be done in order to recreate the enzyme-active site properties as accurately as possible. The work of Himo et al., on the Quantum Chemical Cluster approach, shows that systems that are too small are not suitable to model the chemical activity of enzymes.^21,23^ Second, an IQA analysis is run on a subset of the larger system. This study shows that computing IQA energies of a truncation of the larger system wave-function still results in chemically meaningful REG-IQA results. Using the full-system wave-function is convenient because it keeps as much information as possible about the environment of the enzyme active site that affects the atoms of the lite system. In other words, the topological atoms within the lite system are obtained from the full-system wave-function. Hence, there is no loss in accuracy. On the contrary, running the IQA analysis on the chosen lite system wave-function will lower the reliability of the REG analysis. In order to demonstrate this, Figures 8 and 9 and Table S6 show the REG-IQA results for the HIV-1 protease for the lite system only. Here, the full 133-atom model is used only for the reference geometries but the IQA analysis is employed just on the subset made of 27 atoms. Thus, this wave-function is much smaller than the one used for all previous calculations. Surprisingly, even with an IQA analysis on the lite system, the REG method recovers the most relevant interactions in the peptide hydrolysis. However, the REG values and energy terms calculated are numerically different, showing the qualitative nature of this method. Indeed, the recovered energy gradients are different from the ones obtained in the full system. This is already noticeable by analyzing the shape of the PES in Figure 8, where the 27-atom ab initio calculations (blue) do not recover the actual PES expected from the HIV-1 peptide hydrolysis (black). More specifically, the electrostatic terms are not recovered as well as the exchange–correlation are, due to their strength even at a long range, as expected (Figure 9). This discrepancy is caused by using a completely different wave-function, which is too small and not accurate enough.

#### 3.4.2 Unbiased Selection by Systematic Truncation

In Section S6, we presented a way to consistently assess the REG-IQA method on a specific number of interactions using correlation curves. The better the selection of the most contributing interactions, the nearer to unity is the linear coefficient *m* and vice versa. However, the selection of the atoms was carried out a posteriori and biased by chemical intuition. In theory, such a selection can work in many situations but not always.

We now test a more rigorous way of selecting a relevant subset of atoms again using the HIV-1 protease system. This way involves the same concept of correlation curves represented in eqs S7–S10 but now applied to wave-function energies rather than IQA energies. Let us assume that the translated wave-function energy of a subset of atoms (i.e., the lite wave-function) is fully recovered from the sum of the translated IQA energies at each stationary point of the PES. It is then possible to obtain an idea of the subset of atoms necessary for an accurate REG-IQA analysis. The assumption made above generally holds true within the IQA partitioning scheme, where the atomic integrations deliver decent errors, in the range of units of kJ/mol.

In the quantum chemical cluster approach, it is well known that, when cutting a system, methyl groups should be added at the truncation points.^22^ This is done to represent the dynamical environment of the protein backbone but without introducing too many atoms, which would make DFT calculations too expensive. In the REG-IQA context, the full system is already optimized, and the PES or IRC are already explored. Then, the determined stationary points of the PES are used, and the system is truncated systematically and reasonably. In the specific case of enzymes, removing amino acids or functional groups is the most suitable approach. Only a hydrogen atom is added at the specific point of truncation because the geometry will not be optimized, and thus there is no need to use methyl groups. The hydrogen addition is of course necessary to keep the charge and multiplicity of the original system and to obtain meaningful single-point energy calculations. Note that each hydrogen is added using its van der Waals radius of^59^ ∼1.1 Å and further optimization is not needed in this context (Section S7.1 and Table S7). The full 133-atom system is shown in Figure S9, while snapshots of 10 truncations are shown in Figure S10. In these figures, the reactive center of the system, which is considered to be the WR water (see Figure 2) molecule with the adjacent Asp25, Asp25′, and substrate residues, is kept in all of the truncations to maintain the charge and multiplicity of the system. If for any reason these were removed from it, the single-point energy calculations would not recover the reaction itself.

**Figure 10:**
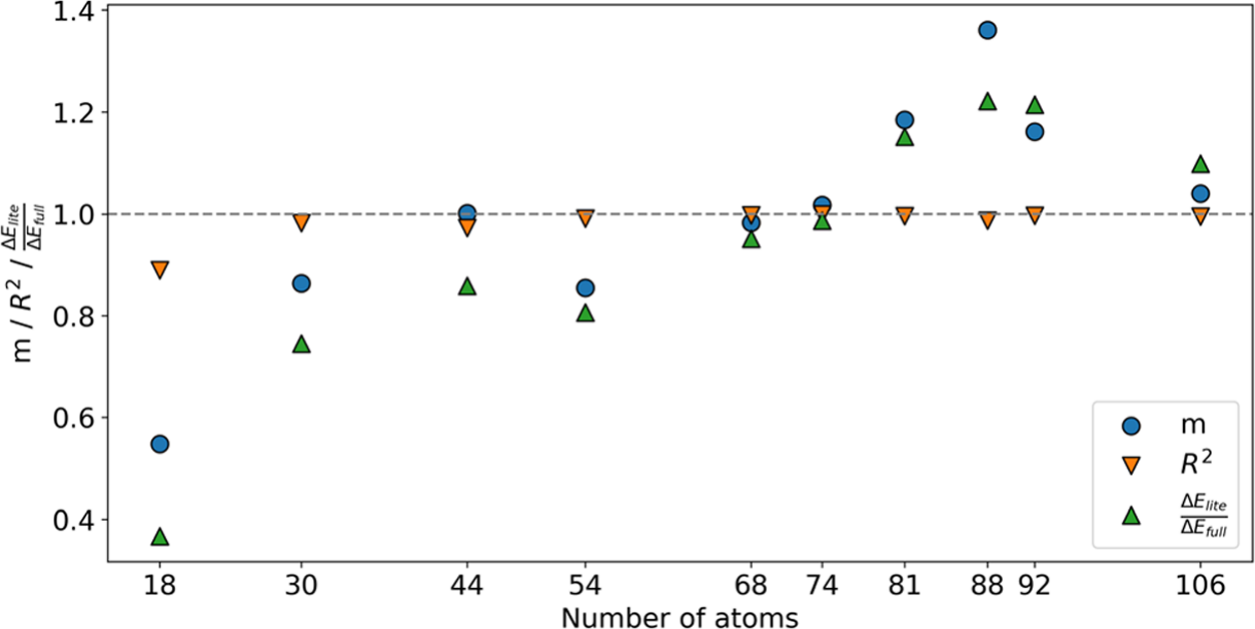
Linear coefficient (*m*), determination coefficient (*R*^2^), and the ratio between the lite system energy of activation (Δ*E*_lite_) and large system energy of activation (Δ*E*_full_) versus the number of atoms at each truncation for the HIV-1 protease system.

Figure S11 shows the comparison between the full 133-atom system PES and the truncated-system PES for each of the 10 truncations. As mentioned earlier, the closer the lite system PES is to the full-system PES, the more accurate the REG-IQA analysis will be. In this case, comparison between the two curves is made by assessing three different values as shown in Figure 10: linear coefficient *m*, coefficient of determination *R*^2^ and the ratio between the Δ*E*s of lite and full systems. The linear coefficient and the Pearson correlation coefficient *R* are obtained as in eqs S8 and S9 but the *E*_IQA_ is here replaced by the wave-function energy of the lite system *E*_WFN,lite_. The coefficient of determination is used instead of *R* because it is more susceptible to systematic changes and more relevant in discriminating between subsets of atoms. The ratio of Δ*E* values (or energies of activation in the case of HIV-1 protease case study) is also a useful criterion to check if a subset of atoms is appropriate for a REG-IQA analysis given its dependency on energy gradients. Overall, the closer the three values  are to unity, the more suitable the subset of atoms will be. Specifically, *R*^2^ checks for the “goodness of fit”, *m* verifies how similar the energies are at each point of the curvature (i.e., how similar are the shapes of the curves), and the ratio of energies confirms that the newly recovered PES is still overall related to the original PES. An example where only two out of three criteria values are met is group *I* (44 atoms). Here, *m* = 1.0 and *R*^2^ is close to 1, while the ratio between the energies of activation is ∼0.8, meaning that the energy gradients between points of the lite system PES are different from the original full-system PES. Hence, the REG-IQA analysis will have different REG values for each partitioned energy term. On the contrary, it can be observed that the original PES is fully recovered when considering group *F* (74 atoms) or *G* (68 atoms). The latter is chosen as an example, and the REG-IQA analysis briefly shown in Table S8 and Figure S12. The overall outcome is that employing a 68-atom wave-function outputs a meaningful REG-IQA analysis at a fraction of the computational cost. Indeed, IQA calculations took ∼2 days per stationary point on eight CPU cores. A test on the cheapest possible integration settings was also carried out for this system. Remarkably, the same qualitative REG-IQA outcome can be obtained at ∼0.8 days per point on eight CPU cores by employing a basin outer angular quadrature of ≤1800 points and a sparse interatomic surface mesh.

## 4 REG-IQA of the HheC System

In this section, all that has been learnt for the HIV-1 protease is applied to the HheC system. The system transition-state coordinates were taken from ref (33). Given that AIMAll can only handle few DFT functionals (namely, B3LYP, PBE, PBE0, LSDA, and M06-2X), the transition state was reoptimized from the given PBE3h/Def2TZVP geometry to an M06-2X/Def2TZVP geometry. Once the imaginary frequency corresponding to the reaction coordinate was obtained, around 30 geometries were generated following the reaction toward the reactants. Geometries up to an energy minimum were then fed into the Ramer–Douglas–Peucker algorithm with an RMSE of 0.25 kJ/mol, thereby obtaining a selection of points (see Figure S13) for the following REG-IQA analysis with systematic truncation.

**Figure 11:**
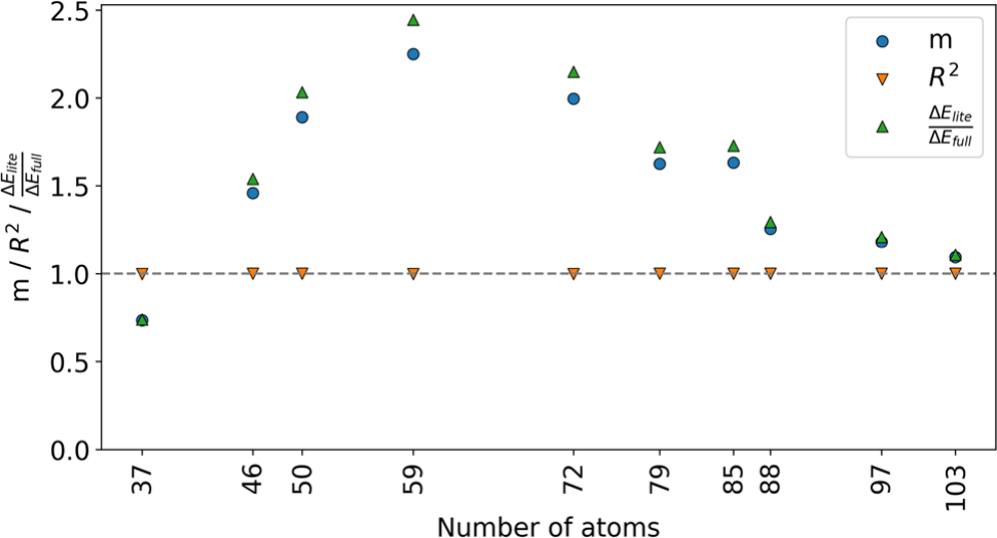
Linear coefficient (*m*), determination coefficient (*R*^2^), and the ratio between the lite system energy of activation (Δ*E*_lite_) and full-system energy of activation (Δ*E*_full_) versus the number of atoms at each truncation for the HheC system.

Figure 11 shows the systematic truncation results. Functional groups and amino acids were removed starting from the outside and moving toward the center of the active site. There is no clear truncation that has all three metrics converging to unity because, most likely, the 112-atom system was already a good minimal subset for the reaction. However, here we show that chemical intuition is recovered even for a subset for which the three error metrics of the systematic truncation do not fully converge to unity but approach it closely.

**Figure 12:**
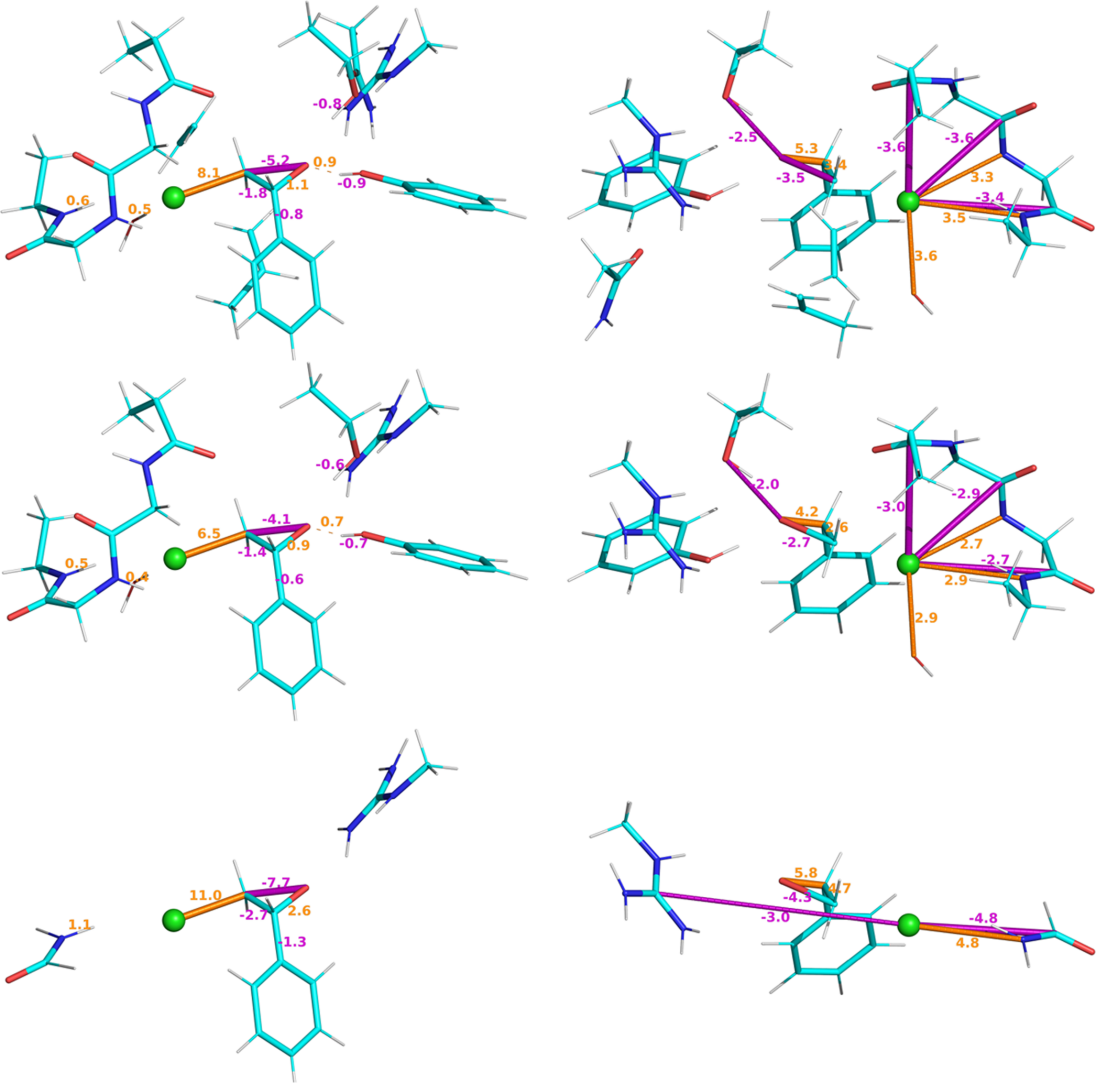
REG-IQA results for the HheC system at different truncation levels. Carbon atoms are in cyan and Cl^–^ is shown as a green sphere. All REG values are shown as a set of dashed cylinders in orange for positive values and magenta for negative ones. Higher REG values are shown with thicker and multiple cylindric dashes, while lower REG values are shown with thinner dashes. The highest positive and negative values determine the scale for the rest of all of the values. (Top) Full HheC 112-atom system: REG*-V*_xc_ (top left) and REG-*V*_cl_ (top right) analysis. Five positive and five negative values are depicted. (Middle) HheC 88-atom truncation system: REG*-V*_xc_ (middle left) and REG*-V*_cl_ (middle right) analysis. Five positive and five negative values are depicted. (Bottom) HheC 37-atom system REG-*V*_xc_ (bottom left) and REG-*V*_cl_ (bottom right) analysis. Three positive and three negative values are depicted. Different orientations of the system are shown for *V*_xc_ and *V*_cl_ interactions to enhance the visual perception of the systems. The static transition-state structure is shown but we should keep in mind that the REG values represent chemical information for a dynamical movement, which is the reaction coordinate from the reactant to the transition state in this case.

REG-IQA results for both *V*_xc_ and *V*_cl_ are presented in Figure 12 using the PyMol-REG^50^ plugin. The full system and two truncations (37 and 88 atoms, respectively) are analyzed to show the potential of the systematic truncation. As stated earlier, no full analysis and description of the REG-IQA is pursued as it is beyond the scope of this study. It is straightforward to see that the REG analysis is fully kept within the 88-atom truncation (middle) for both *V*_xc_ and *V*_cl_ IQA components. Remarkably, for *V*_xc_, the 37-atom truncation shows a fairly good agreement for the main part of the reaction, which is the production of an epoxide through formation of an O–C bond and breaking of the C–Cl bond in the center of the active site. However, this truncation fails at correctly recovering the electrostatics because the Cl anion has no protein backbone to interact with. Similar results have been observed and mentioned earlier (Section 3.4.1). Exchange–correlation is a short-range effect, while electrostatics are mainly long-range. Hence, the systematic truncations need to reflect an environment that considers those effects. However, another important outcome is that, if one seeks mainly breaking–forming bonds patterns, then one can work with smaller truncations and at a much lower computational cost to obtain a relevant REG-IQA analysis. Furthermore, even if only 24 atoms (from 112 to 88) are truncated, then the IQA integrations for each geometry are still twice as fast for 88 atoms than for 112 atoms (see Section S9.2).

## 5 Conclusions

Recently, REG-IQA analyzed the reaction mechanism of the peptide hydrolysis of HIV-1 protease. This new method discovered the well-known “arrow” mechanism by considering exchange–correlation and electrostatic terms^28^ but also other more subtle effects such as “through-space” interactions. This analysis was carried out using the topological energy partitioning called IQA, which is parameter-free and reference-state-free while not depending on the orbitals used. Moreover, IQA works with space-filling atoms. These properties make REG-IQA an accurate, reliable, and minimal method to explore the nature and strength of interactions along a reaction coordinate or any spatial rearrangement of interest.

Despite the advances in computational power, REG-IQA is extremely time-consuming for enzyme modeling. Thus, we developed a strategy here to use REG-IQA routinely. Indeed, for a quantum mechanical system of 133 atoms, which is nowadays considered medium sized, IQA integrations on one single point could take up to an entire CPU month to complete in the past. Here, we propose four strategies to tackle this problem, which are applied to the HIV-1 protease benchmark.

The first strategy is related to the choice of number of grid points for the IQA computation itself. We have shown that using the lowest number of grid points in a sparse mesh yields exactly the same REG-IQA analysis as for higher (default) settings with a 3-fold speed-up in computation for each single-point geometry. However, care should be taken for each system. We recommend default settings if the QTAIM-IQA program AIMAll is used and enough computational resources are available, as each system is unique in its own electron density topology.

The second strategy involves computing a minimum number of points along the reaction coordinate via the Ramer–Douglas–Peucker algorithm.^51−54^ Depending on the desired accuracy, more or fewer geometries can be considered by a user-determined RMSE of tolerance. Considering only 6 out of 11 geometries in the HIV-1 protease reaction yields an RMSE of 0.36 kJ/mol, which means that the REG values are still very reliable. This success almost halves the total computation time.

The third strategy involves a “biased” approach of selecting the number of atoms and interactions for which IQA energies are required. The biased (i.e., based on chemical intuition) selection of a subset of atoms involves extracting information only on specific atoms of the full-system wave-function. This results in an accurate and reliable analysis only if the correct atoms are selected. Here, we show that the interactions involving only 27 atoms out of 133 give the same chemically relevant information (retrieved by REG) as in the 133-atom analysis. These interactions do so at a fraction of the computational cost: from an average of ∼670 CPU hours per geometry for 133 atoms to an average of only ∼67 CPU hours per geometry point for 27 atoms using the same 133-atom wave-function and running on eight CPU cores.

The fourth strategy involves an unbiased selection, i.e., a systematic truncation of the original system into smaller subsets of atoms without invoking chemical decision making. Here, 10 different truncations were made on the HIV-1 protease. Three error metrics  were used to assess the correlation between the energies of the full-system wave-function and the lite system wave-function. It was shown that group *G* (64 original atoms with four added hydrogens) REG-IQA analysis has an almost exact output to that of the 133-atom system. The computational overhead for this approach is ∼49 CPU hours per geometry (∼19 with smallest grid settings). The time needed for system truncation and the single-point energy calculations (i.e., the setup) depends on factors such as the number of truncations explored and the system size. However, this overhead is handsomely compensated by the overall gain in CPU time compared to running IQA calculations on the full system.

Throughout the study, correlation curves were used as an alternative error metric to the recovery error, which increases with the number of numerical integrations. These metrics are not affected by systematic errors as an RMSE is and are helpful for comparing shapes (i.e., energy gradients) of different PESs. Thus, these metrics are more meaningful within the context of the construction of the “lite” version of REG-IQA. Application of the methodology has also been shown to work with the HheC system. In this system, the systematic truncation approach is less efficient than for the HIV-1 protease system due to long-range electrostatics being a key component of the reaction itself due to the presence of a Cl anion. However, from a covalency point of view, the reaction’s chemical insight is recovered even with the smallest truncation and at dramatically lower computational cost.

In conclusion, REG-IQA has been demonstrated to be an accurate and insightful method for the HIV-1 protease peptide hydrolysis benchmark and for the haloalcohol dehalogenase HheC test system. Thanks to the current work, the computational cost of such an analysis has been drastically reduced. Hence, it is now computationally feasible to apply REG-IQA routinely to other and larger systems of interest.

## Supporting Information Available

Some REG-IQA background, figures, and tables for the HIV-1 protease systems and the HheC systems, REG-IQA analyses, an IQA computational expense account, and RDP algorithm notes (PDF)
