# Supplementary material for: How to Compute Atomistic Insight in DFT Clusters: The REG-IQA Approach
Source: J Chem Inf Model. 2023 Jul 10;63(14):4312–27. doi: 10.1021/acs.jcim.3c00404 (PMC10369488; doi:10.1021/acs.jcim.3c00404)
Supplement: Supplementary file 1 — ci3c00404_si_001.pdf [file ci3c00404_si_001.pdf]

Supporting Information

How to Compute Atomistic Insight in DFT clusters: the REG-IQA Approach

Fabio Falcioni and Paul LA Popelier

Department of Chemistry, University of Manchester, Oxford Road, Manchester M13 9PL, Great Britain

*Corresponding Author: paul.popelier@manchester.ac.uk, +44 161 3064511

Table of Contents

1 Background Additional Information 2
1.1 Note on IQA computational expense 2
1.2 Interacting Quantum Atoms (IQA) 2
1.3 Relative Energy Gradient (REG) 3
2 REG Stationary Points Dependency 4
3 IQA Grids Benchmark 5
4 Ramer-Douglas-Peucker (RDP) algorithm 5
5 Selection of a subset of atoms 9
6 Correlation Curves as an Alternative Error Metric 10
7 System Truncation 15
7.1 Hydrogen Addition 18
7.2 HIV-1 Protease 68 atoms 19
8 IQA Timings for HIV-1 Protease 20
9 HheC system 21
9.1 RDP algorithm 21
9.2 Timings 21

1 Background Additional Information

1.1 Note on IQA computational expense

In the case of the 2018 original HIV study, the total number of integrations is:

$$\left[133 + \frac{133(133-1)}{2}\right] 11 = 98021 \quad \text{(S1)}$$

where n = 133 and N_{steps} = 11 or eleven geometries of the PES. Considering that these integrations involve a six-dimensional integral over the finite volume of each atom, these are very time-consuming. Moreover, IQA depends on the wave-function obtained from QM calculations. Different levels of theory (e.g., HF, DFT, CCSD, MP2 with different basis sets) change the number of Gaussian primitives that make up the wave-function.

1.2 Interacting Quantum Atoms (IQA)

Note that the deformation energy E^{A}_{def} is defined as a difference between $E^{A}_{intra,in-system}$ and $E^{A}_{intra,vacuo}$, which is more meaningful when it comes to molecular displacement, as encountered in reactions or in van der Waals complexes. Moreover, it has been recently demonstrated that $E^{A}_{def} = E^{A}_{sh} + E^{A}_{ct}$, where E^{A}_{sh} is the intra-atomic steric hindrance while E^{A}_{ct} is the intra-atomic charge transfer. This makes E^{A}_{sh} a more faithful quantifier. However, this quantity will not be analysed in this study but will prove to be helpful in future analyses. The chemical information obtained from the V^{AB}_{cl} terms can be derived from

$$V^{AB}_{cl} = V^{AB}_{ct} + V^{AB}_{pl} \quad \text{(S2)}$$

where V^{AB}_{ct} is the monopolar/charge-transfer term obtained by using a simple Coulomb equation (eq S3) between the total (net) charge of each atomic basin (q^A and q^B) and their internuclear distance r_{AB}, while V^{AB}_{pl} consists of polarisation energy that the whole system induces to the interaction between atoms *A* and *B* when all monopolar energy has been removed (eq S4),

$$V^{AB}_{ct} = \frac{q^A q^B}{r_{AB}} \quad \text{(S3)}$$

$$V^{AB}_{pl} = V^{AB}_{cl} - V^{AB}_{ct} \quad \text{(S4)}$$

This separation gives a more detailed and intuitive understanding of the classical electrostatic energy recovered within IQA.

1.3 Relative Energy Gradient (REG)

As a general example for explaining the REG methodology we quote the Morse potential, which describes the energy of two atoms moving from infinity to short-range or the other way around and is shown in eq S5,

$$E(r) = D_e\left[1 - e^{-a(r-r_e)}\right]^2 - D_e, \quad \text{(S5)}$$

which can be rewritten as

$$E(r) = D_e\left(e^{-2a(r-r_e)} - 2e^{-a(r-r_e)}\right) \quad \text{(S6)}$$

where D_e is the well depth, r_e the equilibrium bond distance between the atoms and a is ultimately related to the spring constant in Hooke's model in the regime very close to the energy minimum. Note that D_e is subtracted in eq S5 because the zero-reference of the potential energy corresponds to the two atoms at infinite distance. Equation S6 shows a combination of a repulsive potential (first term), which governs the left side of the Morse potential, and an attractive one (second term), which regulates the right side. This behaviour follows from Figure S1. This analysis is useful to understand what happens in many PESs and is thus universal for the REG-IQA framework. Hence, a single PES depends on specific interactions at different stages of the dynamical evolution.

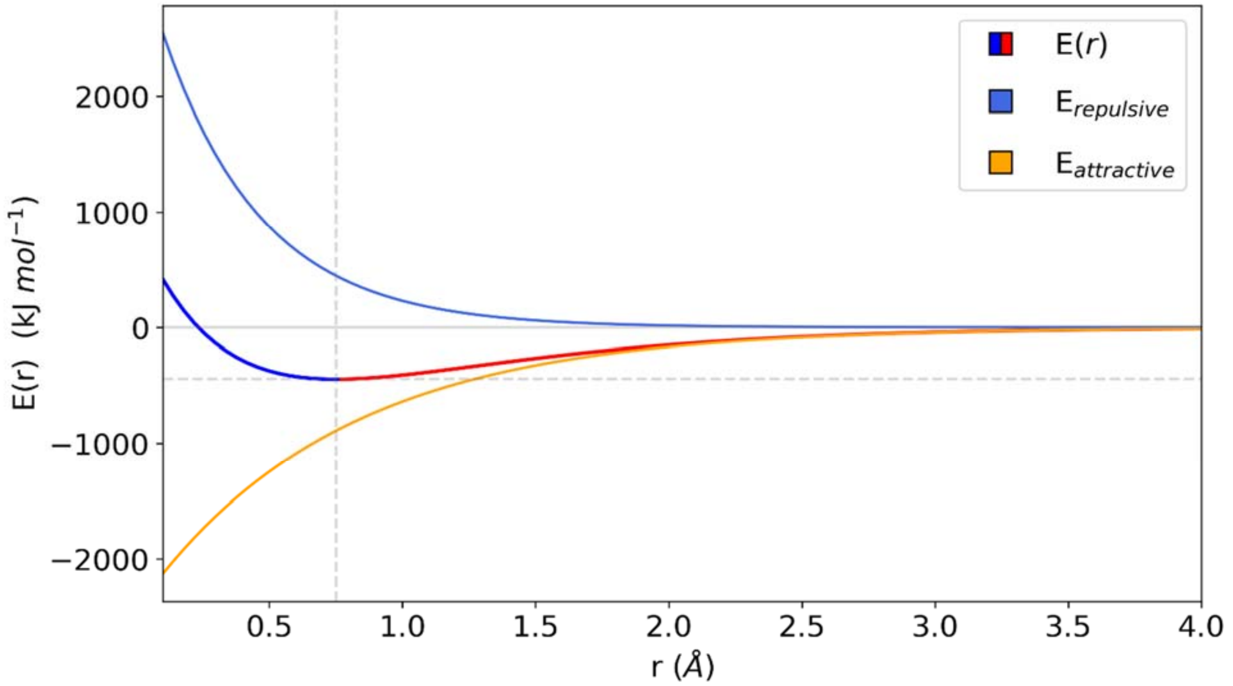

Figure S1. Morse potential for two hydrogen atoms forming a hydrogen molecule. The blue and red curves represent the combination of the repulsive and attractive potential shown in eq S6, and are separated by an energy minimum at 0.74 Å.

2 REG Stationary Points Dependency

Table S1. HIV protease REG values for the complete system: (left) V_{xc} and (right) V_{cl}.

TERM	REG	R
V_{xc}(c58,o66)	-4.0	-0.91
V_{xc}(o67,h100)	-3.8	-0.97
V_{xc}(c35,o36)	-1.9	-0.96
V_{xc}(o37,h99)	-1.0	-0.99
V_{xc}(o66,o67)	-0.8	-0.99
V_{xc}(o66,h101)	1.1	0.94
V_{xc}(c35,o37)	1.5	0.96
V_{xc}(o66,h99)	1.8	0.98
V_{xc}(c58,o67)	2.4	0.96
V_{xc}(o36,h100)	3.7	0.97

TERM	REG	R
V_{cl}(o67,h100)	-6.7	-0.96
V_{cl}(c35,o36)	-5.3	-0.97
V_{cl}(c35,h100)	-3.3	-0.96
V_{cl}(c58,o66)	-3.1	-0.96
V_{cl}(o37,h99)	-2.8	-0.99
V_{cl}(c58,h100)	2.4	0.98
V_{cl}(c35,h99)	2.9	1.00
V_{cl}(c58,n59)	6.1	0.77
V_{cl}(o36,h100)	6.4	0.95
V_{cl}(c58,o67)	7.6	0.91

Table S2. HIV protease full system REG values: (left) V_{xc} and (right) V_{cl} on points (1,3,5,7,9,11) of the original potential energy surface.

TERM	REG	R
V_{xc}(c58,o66)	-4.9	-0.95
V_{xc}(o67,h100)	-3.8	-0.96
V_{xc}(c35,o36)	-1.9	-0.95
V_{xc}(o37,h99)	-1.1	-0.99
V_{xc}(o66,o67)	-0.8	-0.99
V_{xc}(o66,h101)	1.3	0.97
V_{xc}(c35,o37)	1.5	0.95
V_{xc}(o66,h99)	2.0	0.99
V_{xc}(c58,o67)	2.5	0.96
V_{xc}(o36,h100)	3.6	0.95

TERM	REG	R
V_{cl}(o67,h100)	-6.6	-0.95
V_{cl}(c35,o36)	-5.5	-0.97
V_{cl}(c35,h100)	-3.1	-0.95
V_{cl}(c58,o66)	-3.1	-0.95
V_{cl}(o37,h99)	-2.9	-0.99
V_{cl}(c35,o37)	2.4	0.91
V_{cl}(c35,h99)	3.0	1.00
V_{cl}(o36,h100)	6.0	0.93
V_{cl}(c58,n59)	8.7	0.91
V_{cl}(c58,o67)	8.7	0.92

Table S3. HIV protease full system REG values: (left) V_{xc} and (right) V_{cl} on points (1,5,8,9,10,11) of the original potential energy surface.

TERM	REG	R
V_{xc}(c58,o66)	-4.2	-0.89
V_{xc}(o67,h100)	-3.9	-0.96
V_{xc}(c35,o36)	-2.0	-0.96
V_{xc}(o37,h99)	-1.0	-0.98
V_{xc}(o66,o67)	-0.8	-0.98
V_{xc}(o66,h101)	1.1	0.93
V_{xc}(c35,o37)	1.5	0.96
V_{xc}(o66,h99)	1.9	0.97
V_{xc}(c58,o67)	2.5	0.96
V_{xc}(o36,h100)	3.8	0.96

TERM	REG	R
V_{cl}(o67,h100)	-6.9	-0.96
V_{cl}(c35,o36)	-5.4	-0.97
V_{cl}(c35,h100)	-3.4	-0.96
V_{cl}(c58,o66)	-2.9	-0.95
V_{cl}(o37,h99)	-2.8	-0.99
V_{cl}(c58,h100)	2.4	0.98
V_{cl}(c35,h99)	2.8	1.00
V_{cl}(c58,n59)	6.6	0.76
V_{cl}(o36,h100)	6.6	0.94
V_{cl}(c58,o67)	8.1	0.90

3 IQA Grids Benchmark

Here we show results in connection with Section 3.2 of the main manuscript. The default settings of the program AIMAll and its custom settings (mesh=sparse, outer angular quadrature = GS1) are compared for the V_{xc} and V_{cl} energy terms. Table S4 shows the REG values of custom setttings and Table S3 those obtained with the defaultsettings. The results obtained using computationally cheap customised integration grids perfectly match those obtained by the default integration settings for the HIV-1 protease system of 133 atoms.

Table S4. HIV protease REG values: (left) complete system V_{xc} and (right) V_{cl}. Custom integration settings for AIMAll were applied, with sparse mesh and outer angular quadrature GS1 (≤ 1800 grid points).

TERM	REG	R
Vxc(c58,o66)	-4.2	-0.89
Vxc(o67,h100)	-3.9	-0.96
Vxc(c35,o36)	-2.0	-0.96
Vxc(o37,h99)	-1.0	-0.98
Vxc(o66,o67)	-0.8	-0.98
Vxc(o66,h101)	1.1	0.93
Vxc(c35,o37)	1.5	0.96
Vxc(o66,h99)	1.9	0.97
Vxc(c58,o67)	2.5	0.96
Vxc(o36,h100)	3.8	0.96

TERM	REG	R
Vcl(o67,h100)	-6.9	-0.96
Vcl(c35,o36)	-5.4	-0.97
Vcl(c35,h100)	-3.4	-0.96
Vcl(c58,o66)	-2.9	-0.95
Vcl(o37,h99)	-2.8	-0.99
Vcl(c58,h100)	2.4	0.98
Vcl(c35,h99)	2.8	1.00
Vcl(c58,n59)	6.6	0.76
Vcl(o36,h100)	6.6	0.94
Vcl(c58,o67)	8.1	0.90

4 Ramer-Douglas-Peucker (RDP) algorithm

We briefly discuss the application of RDP to PES together with some examples in Figure S2-S3. The main point to explain is the nature of the original RDP tolerance epsilon (ε) in the context of PESs. This value in RDP is an actual distance in a geometrical space (e.g. pixels versus pixels of an image). During the recursive RDP iterations ε is strictly compared to the maximum perpendicular distance of a point to the polyline, which has the same units as ε. Thus, whichever the units of ε are, the algorithm will work correctly if the perpendicular distance has its same units. This is what makes the algorithm applicable to PESs (i.e. y=energy, x=distance) even if epsilon does not have a physically relevant unit.

As an example, the IRC of a simple S_N2 reaction is used to benchmark the RDP algorithm. Details on the QM calculations are omitted because they are not relevant in the context of RDP.

Figure S2 shows the effect of the RDP algorithm at different RMSE threshold values. It is clear that with an RMSE of ~0.7 kJ/mol, the potential energy surface (PES) is trimmed from 31 points to only 9 points. Given that the error is only ~2% (0.7 kJ/mol to ~35 kJ/mol) of the maximum energy difference (the energy of activation from reactants to transition state), it is safe to assume that the REG-IQA analysis on the 9 points is reliable.

In Figure S3, the same first-principles PES is defined with 50 and 100 points. As mentioned earlier, the RDP algorithm acts independently of the number of points of the initial polyline. Nevertheless, the polylines that were recovered at the same RMSE of tolerance (0.1 kJ/mol) are very similar. This means that there is not a specific number of points necessary for the RDP algorithm to operate on. However, the user should be aware of the computational time to obtain the original PES. Indeed, a 100-points PES scan is computationally much more demanding than a 50-points one (also depending on the system size). On the contrary, fewer starting points would make the RDP algorithm too severe while reducing the number of points, leading to an unreliable REG-IQA analysis. Note that Figure S2 and S3 show a different value of RMSE compared to the chosen RMSE of confidence of 0.1 kJ/mol. That is because the algorithm is jumping between a polyline that has an RMSE lower than 0.1 kJ/mol to another one that has a higher one, and therefore chooses the one lower than the chosen threshold.

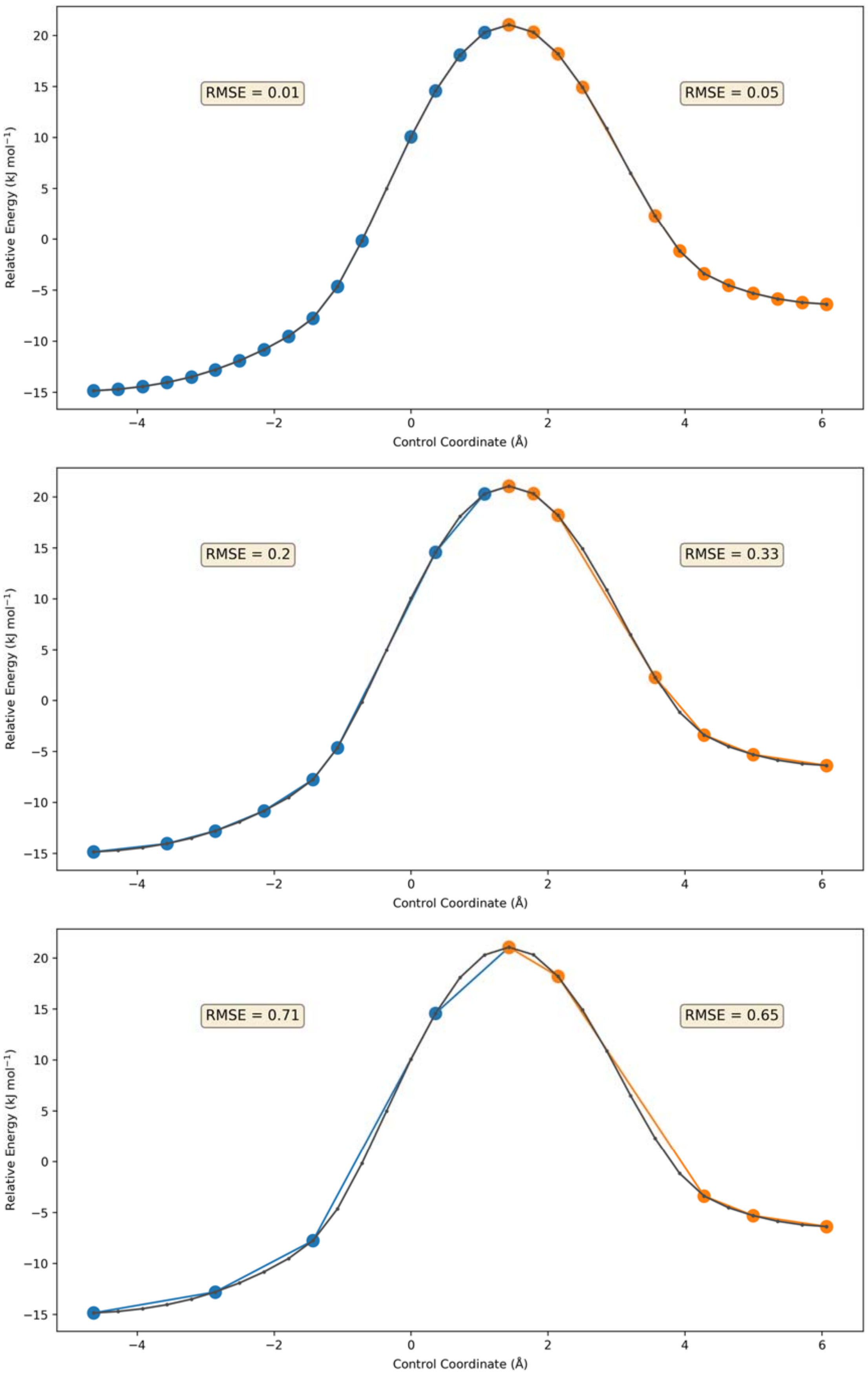

Figure S2. S_N2 PES with chosen RMSE confidence of (top) 0.1, (middle) 0.5 and (bottom) 1.0 kJ/mol. Each PES is divided into segments (blue and orange), and near each minimised segment the relative RMSE is shown. The grey polyline represents the *ab initio* PES (31 points).

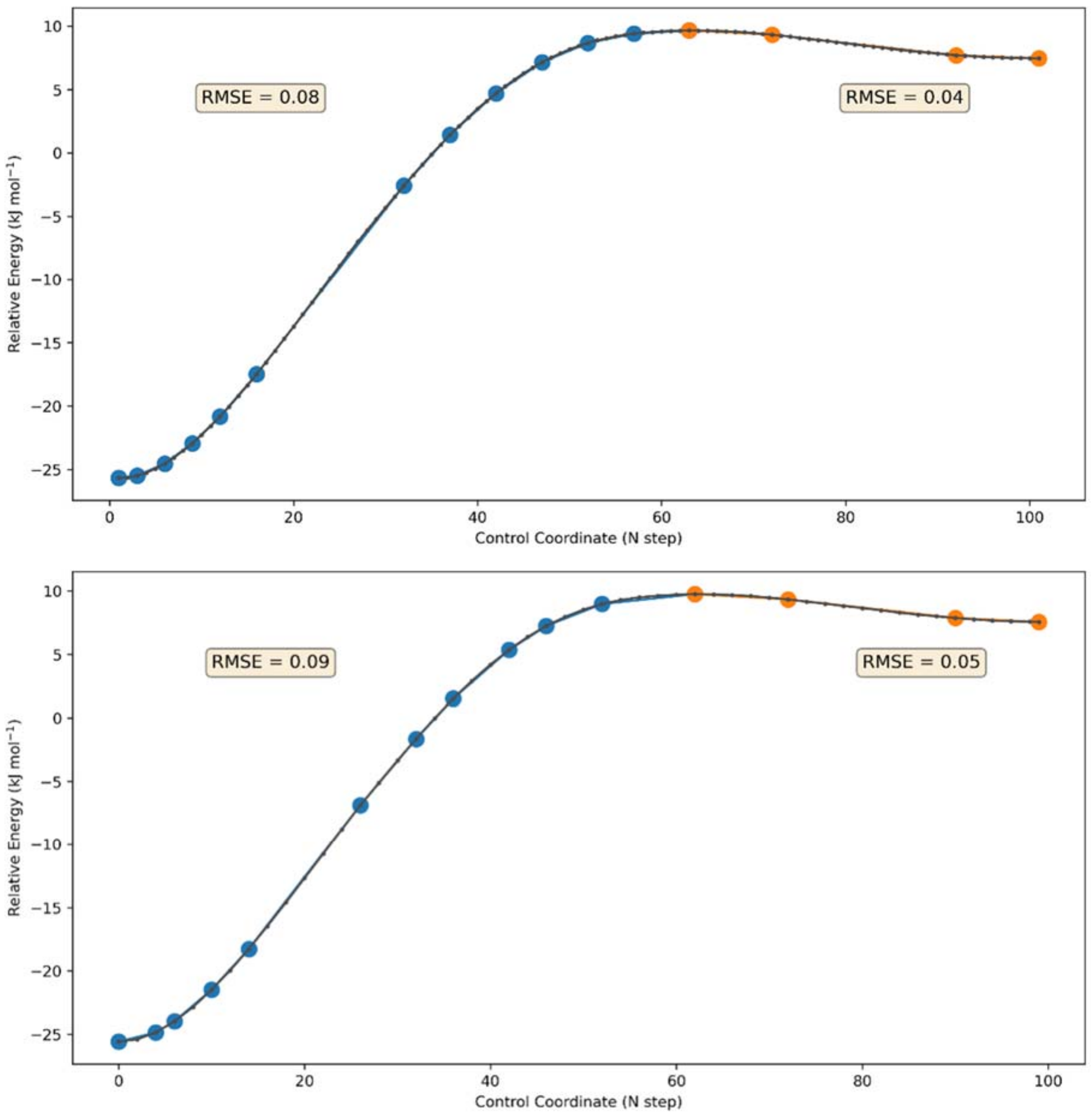

Figure S3. Example of many-points PES with chosen RMSE tolerance of 0.1 kJ/mol. Each PES represents only one segment (blue), and the relative RMSE is shown near each minimised curve. The grey polyline represents the first-principles PES with (top) 100 points and (bottom) 50 points.

5 Selection of a subset of atoms

Table S5. HIV protease full system REG values: (left) V_{xc} and (right) V_{cl} on points (1,5,8,9,10,11) of the original PES.

REG step	1	5	8	9	10	11
Vcl(o58,o67) – Large system	-0.798	-0.915	-1.015	-1.018	-1.021	-1.020
Vcl(o58,o67) – Lite system	-0.798	-0.915	-1.015	-1.018	-1.021	-1.020
Vxc(o58,o67) – Large system	-0.284	-0.304	-0.338	-0.344	0.347	0.347
Vxc(o58,o67) – Lite system	-0.284	-0.304	-0.338	-0.344	0.347	0.347

Table S6. HIV protease lite system (small wave-function) REG values: (left) V_{xc} and (right) V_{cl} (right) on points (1,5,8,9,10,11) of the original PES.

TERM	REG	R
V_{xc}(c58,o66)	-5.3	-0.98
V_{xc}(o67,h100)	-4.9	-0.95
V_{xc}(c35,o36)	-2.9	-0.95
V_{xc}(o37,h99)	-1.0	-0.98
V_{xc}(o66,o67)	-0.9	-0.99
V_{xc}(c9,o11)	1.1	0.99
V_{xc}(o66,h101)	1.7	1.00
V_{xc}(o66,h99)	1.8	1.00
V_{xc}(c58,o67)	3.2	0.97
V_{xc}(o36,h100)	4.8	0.96

TERM	REG	R
V_{cl}(o67,h100)	-7.7	-0.96
V_{cl}(c35,o36)	-4.9	-0.99
V_{cl}(c35,h100)	-3.5	-0.94
V_{cl}(c58,o66)	-2.9	-0.88
V_{cl}(c9,o10)	-2.5	-0.98
V_{cl}(c35,h99)	3.1	0.97
V_{cl}(c35,o37)	4.4	0.97
V_{cl}(o36,h100)	8.0	0.93
V_{cl}(c58,n59)	8.5	0.93
V_{cl}(c58,o67)	9.5	0.98

6 Correlation Curves as an Alternative Error Metric

The *recovery error*, defined as the RMSE between the true system's energy and the sum of IQA "recovered" energies, is a valuable metric to assess the REG-IQA method quantitatively. However, it has been shown that this value increases with the system size. This value seems very large in the case of the HIV-REG lite system analysis (around 33 kJ/mol), although the latter still outputs relevant REG rankings and agrees with the full-system calculations. Indeed, having more atoms in a system means that many more IASs are involved, which leads the clocking up of integration errors (see deviations from zero in equation 4 of main manuscript). However, these errors are not relevant considering that they make up a very low percentage of the total absolute energy of a system, which is usually in the range of thousands of kJ/mol. A better way to assess the capabilities of REG-IQA is to remove any bias present due to the IQA energy integrations. Specifically, one can use a familiar concept that is the foundation of the REG analysis: correlation curves. Correlating the energy obtained from the original unpartitioned QM calculations with the sum of the partitioned IQA energies at each stationary point gives an unbiased metric for the REG-IQA method. A simple least-squares linear regression is performed on the stationary point energies (E_{WFN}) translated over their mean as in the actual REG analysis. In contrast to the REG analysis on individual IQA terms (see equation 16 of main manuscript), only a single least-squares regression over the total sum of IQA energies (E_{IQA}) is performed at each step of the control coordinate. The key equation employed is

$$E_{IQA}(s) = m \cdot E_{WFN}(s) + c \qquad \text{(S7)}$$

where m is the linear coefficient, c the intercept and s the control coordinate, that is, the dynamic change of the system. Every IQA sum is fitted over M data points of the control coordinate s relative to the analysed curve. The linear coefficient m is obtained as

$$m = \frac{\sum_s^M \left(E_{IQA}(s) - \bar{E}_{IQA}\right)\left(E_{WFN}(s) - \bar{E}_{WFN}\right)}{\sum_s^M \left(E_{WFN}(s) - \bar{E}_{WFN}\right)^2} \qquad \text{(S8)}$$

while the Pearson's correlation coefficient becomes

$$R = \frac{\sum_s^M \left(E_{IQA}(s) - \bar{E}_{IQA}\right)\left(E_{WFN}(s) - \bar{E}_{WFN}\right)}{\sqrt{\sum_s^M \left(E_{IQA}(s) - \bar{E}_{IQA}\right)^2}\sqrt{\sum_s^M \left(E_{WFN}(s) - \bar{E}_{WFN}\right)^2}} \qquad \text{(S9)}$$

Suppose the R coefficient is close to the value of one. In that case, it means that the IQA integrations (both intra-atomic and interatomic) correlate well to the actual *ab initio* energies and that the ensuing REG analysis will be reliable. Also, the closer the linear coefficient is to 1 for any PES segment, the better the IQA energies are recovering the full PES. This is an immediate consequence of equation S8. In the best-case scenario, where each IQA summed energy translated over the energy mean equals the wave-function energy, the calculated m will be equal to 1 (equation S8). Indeed, thanks to the additive property of REG, when all

REG values of a segment are summed together, a value of 1 is obtained if no errors are present due to integrations,

$$m = \frac{\sum_s^M \left(E_{WFN}(s) - \bar{E}_{WFN}\right)^2}{\sum_s^M \left(E_{WFN}(s) - \bar{E}_{WFN}\right)^2} = 1 \qquad when \quad E_{IQA}(s) = E_{WFN}(s) \qquad \text{(S10)}$$

Note that this kind of approach is still giving a quantitative idea on the reliability of the REG-IQA method, even if we are correlating energy differences rather than absolute energies. The correlation curves as presented can also be applied to the lite system when using the full wave-function. Indeed, *if the correlation between the IQA energies and the large system wave-function energies is adequate, it means that most of the relevant intra- and interatomic interactions have been considered throughout the REG analysis*. In other words, even if fewer interactions are considered, a reliable REG-IQA analysis is obtained as long as a balance of favourable and unfavourable interactions is maintained. In contrast, this error metric will not be insightful, although it will technically work, for the lite system corresponding to a small wave-function because the QM PES is not representative of the chemical reaction considered and the IQA integrations did not recover the energy gradients of the real system properly, as mentioned earlier.

Representative graphs and correlation curves for the HIV-1 Protease case study are shown in Figure S4 to demonstrate the concept of correlation curves as an error metric. The first comparison is made between (a) and (d), where the IQA recovered energies are in favourable agreement with the QM calculated energies. However, each stationary point is recovered differently, and there appear to be more IQA energy fluctuations in the 11-steps graph (a) than in the 6-steps graph (c). This is confirmed by looking at the corresponding correlation curves (b) and (d), where a slightly stronger correlation for the latter is observed. Both approaches show that IQA recovers the reaction trend despite the always present integration errors, which are accounted for by the translation over the energy averages. Figure S4 (e) shows the HIV-REG lite system (27 atoms) using the large wave-function. It is observed that IQA is not fully recovering the wave-function energy as the two PES are not overlapping well. This is expected because only 729 out of the possible 17,689 energies of the 133-atom systems are considered. Nevertheless, the correlation between the two energies is still strong (R = 0.977), and the linear coefficient is close to 1 (m = 1.229) as shown in panel (f), explaining that the interactions considered for the REG analysis are those that most contribute to the shape of the PES. Finally, panels (g) and (h) shows the PES and the correlation curve for the HIV-REG lite system calculated considering the small wave-function of 27 atoms. Although the results seem reasonable due to a surprisingly good correlation between the IQA energy and the WFN energy, it must be noticed that the PES is not representative of the HIV-1 peptide hydrolysis. Indeed, the hypothetical ΔE of activation for this smaller system is around 58.7 kJ/mol, which is different from the 66.5 kJ/mol obtained from the 133-atom PES. Moreover, this PES in Figure S4 (g) does not have two clear stationary points (i.e. reactant and transition state) as in the true full-system PES. As examples for correlation curves, two extreme scenarios of a wrong selection of the subset of atoms for HIV-REG lite system are presented in Figures S5-S8.

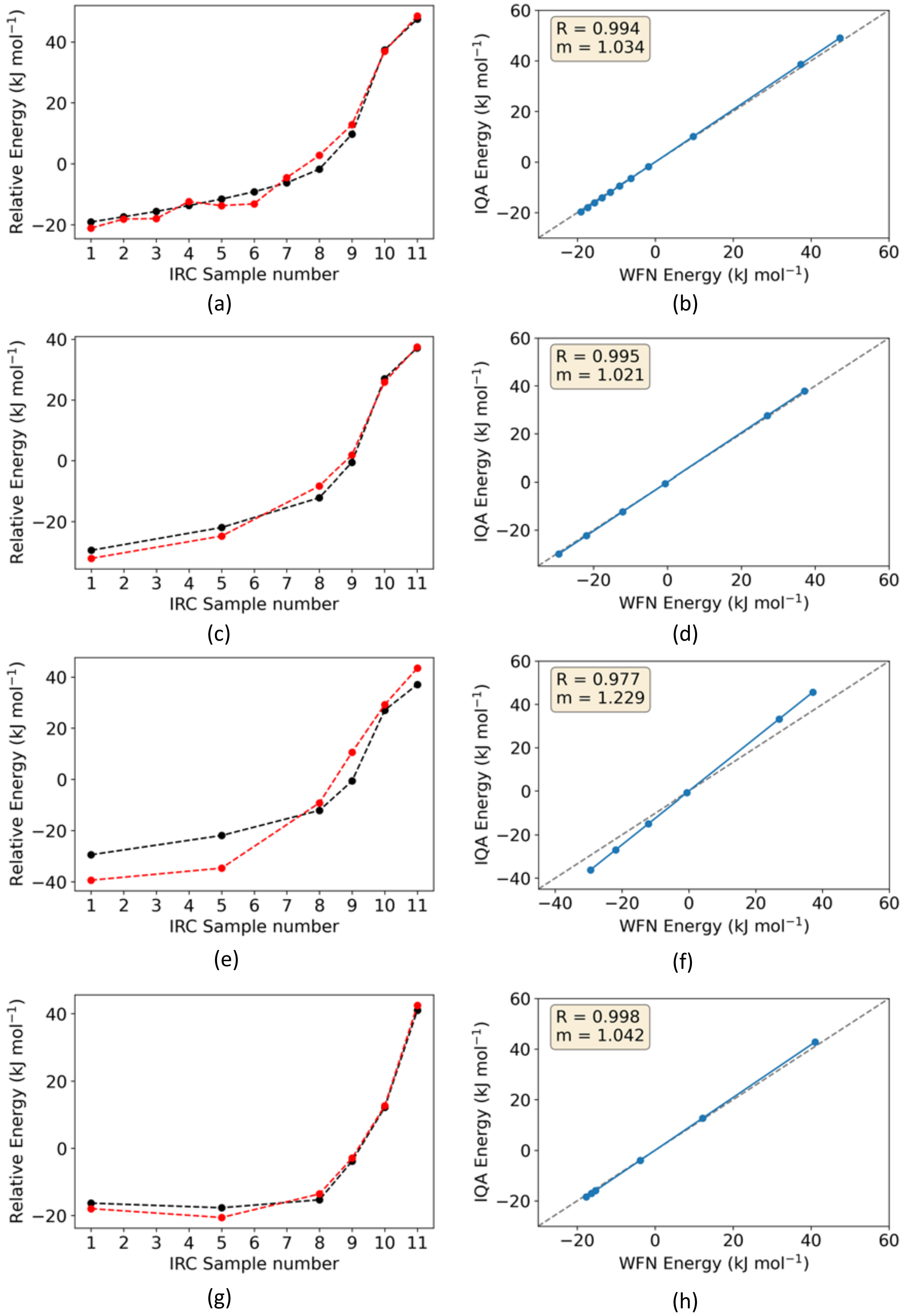

Figure S4. Wave-function PES (black) and IQA recovered PES (red) for the HIV system translated over the relative energy mean for the (a)(b) full and 11 geometries, (c)(d) full and 6 geometries, (e)(f) lite with large wave-function and 6 geometries, and (g)(h) lite with small wave-function and 6 geometries.

Two extreme scenarios of a wrong selection of the subset of atoms for the HIV-REG lite system are presented as examples: one subset with very few atoms (Figures S5-S6) and one with irrelevant atoms (Figures S7-S8). These extreme cases show how correlation curves can be used to understand if relevant atoms (and thus their interactions) were correctly or incorrectly chosen for the IQA integrations in a biased approach (as described in the main text). Figure S6 (a) shows the PES recovered with the sum of 36 (=6x6) IQA energy terms and the wave-function energy of the 133-atom system. Figure S6 (b) represents the correlation curve between the two types of energies. We observe an acceptable correlation but a very high linear coefficient *m*, which is the sum of the 36 REG values of the considered interactions. This high value confirms that strong interactions are considered for that specific segment of the PES but too few to represent the wave-function energy gradients correctly.

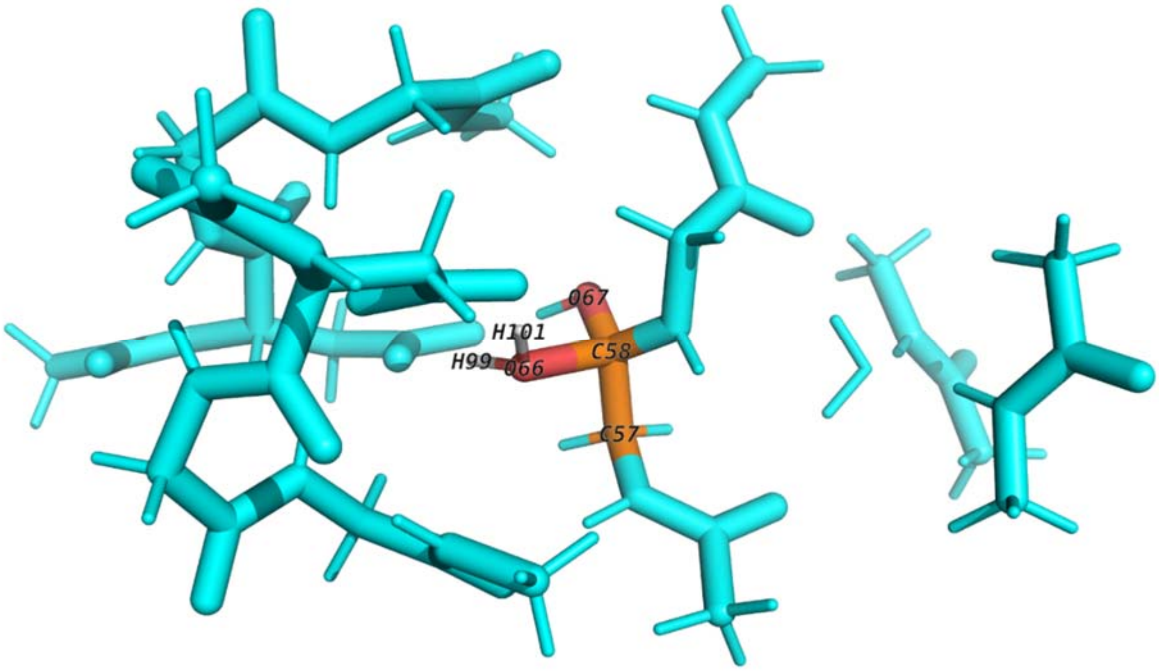

Figure S5. HIV-1 protease active site 133-atom model represented by a lite system that is too small. The lite system is marked in orange with black labels and the rest in cyan. The lite system consists of 6 atoms on which the REG-IQA analysis was performed.

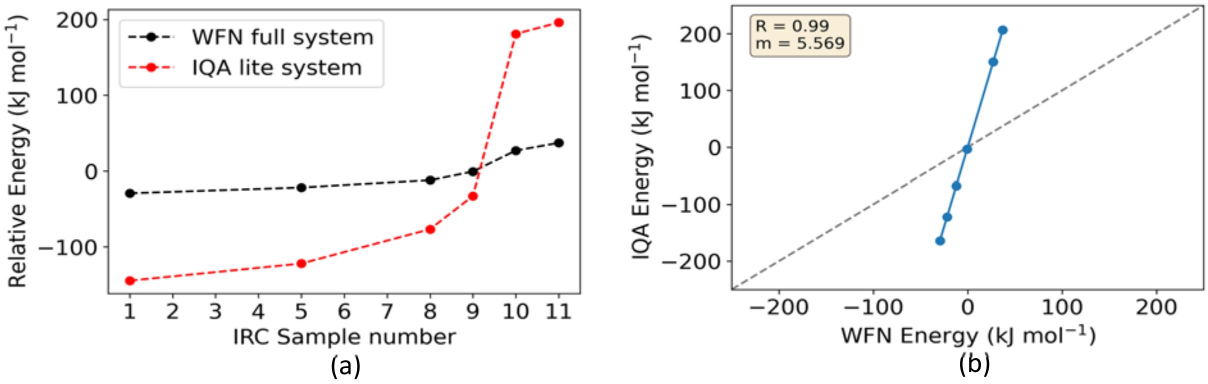

Figure S6. (a) Wave-function PES and IQA PES comparison for a 6-atoms lite system, and (b) relative correlation curve.

Figure S8 (a) shows the PES recovered with the sum of the IQA energies of 24 atoms (depicted in Figure S7) the wave-function energy of the 133-atom system. In this case, there is no good correlation between wave-function energy and IQA energies (Figure S8 (b)). Indeed, given the trend of the original PES, which goes from lower to higher energy, similar behaviour from the recovered IQA PES is expected to occur. However, this does not occur due to the selection of primarily atoms that are irrelevant to the reaction mechanism. In other words, to ensure the atom selection is correct, it is likely that the sum of the REG values of the intra-atomic and interatomic interactions is close to unity, given the additive property of REG.

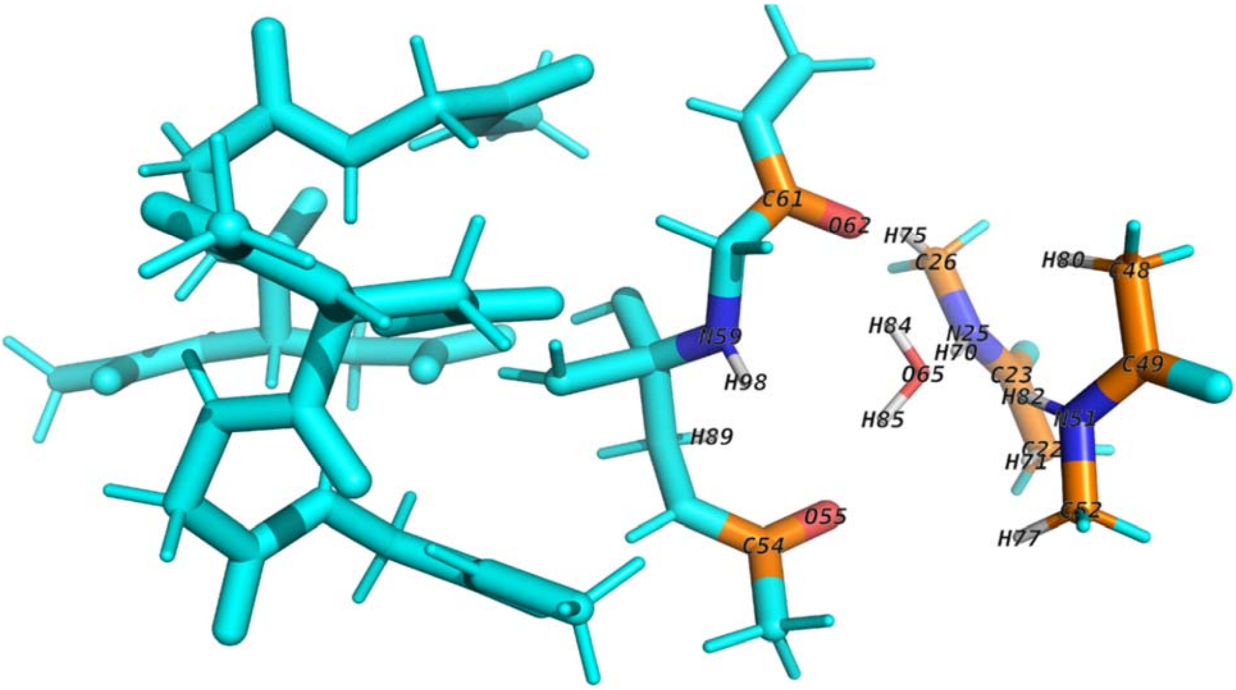

Figure S7. HIV-1 protease active site 133-atom model represented by a lite system that consists of irrelevant atoms. The lite system is marked in orange with black labels and the rest in cyan. The lite system consists of 24 atoms on which the REG-IQA analysis was performed.

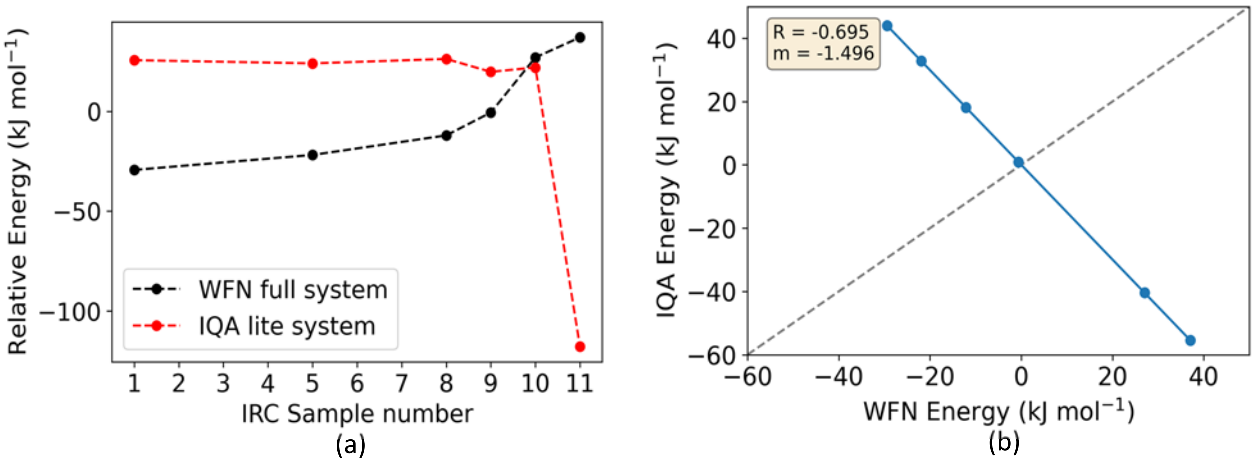

Figure S8 (a) Wave-function PES and IQA PES comparison for 24-atoms lite system, and (b) relative correlation curve.

7 System Truncation

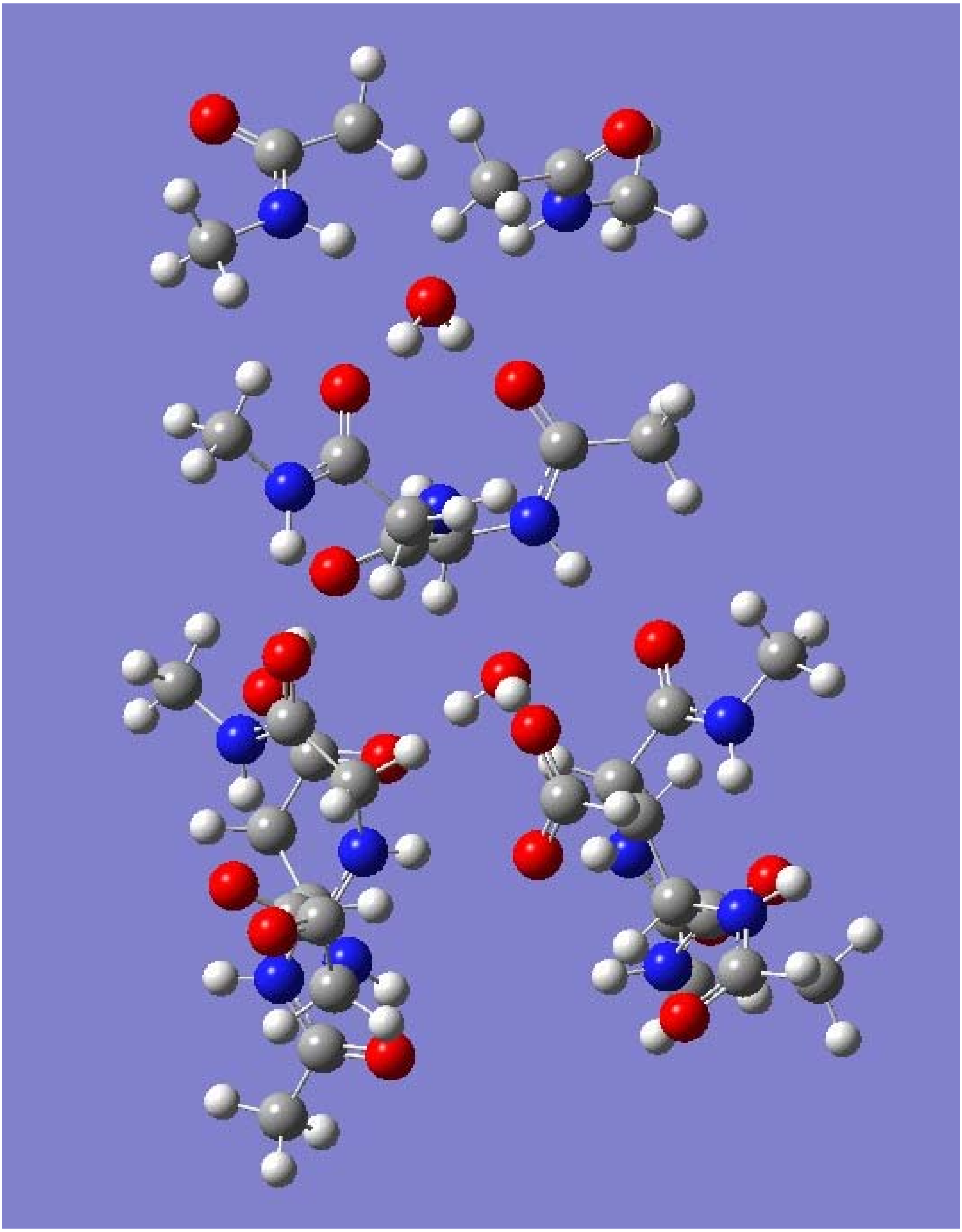

Figure S9. Full HIV-1 Protease original system of 133 atoms.

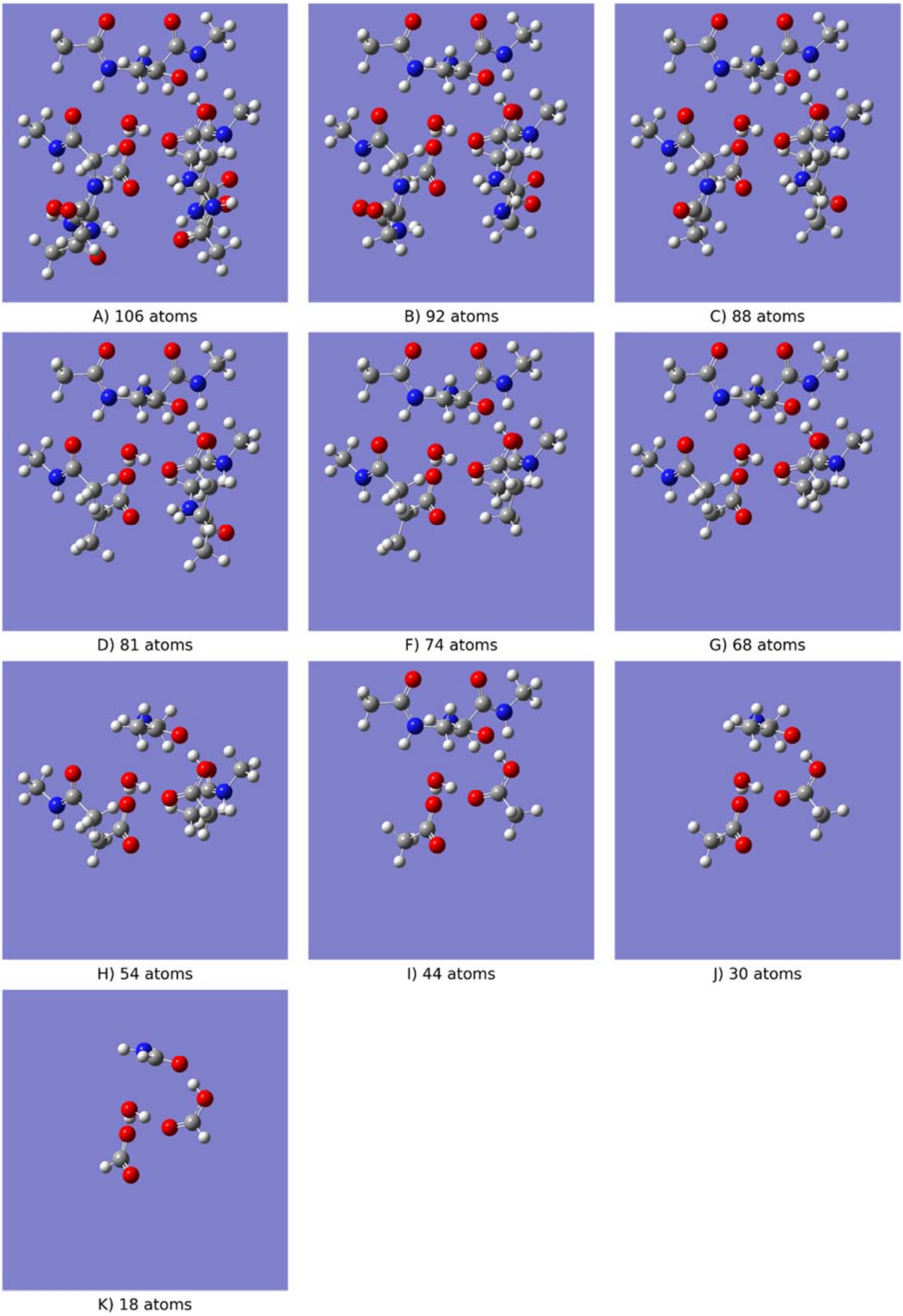

Figure S10. Snapshots of the ten truncations of HIV-1 Protease, used for single-point energy calculations of the reactants. The same truncations were carried out for all the other geometries along the intrinsic reaction coordinate.

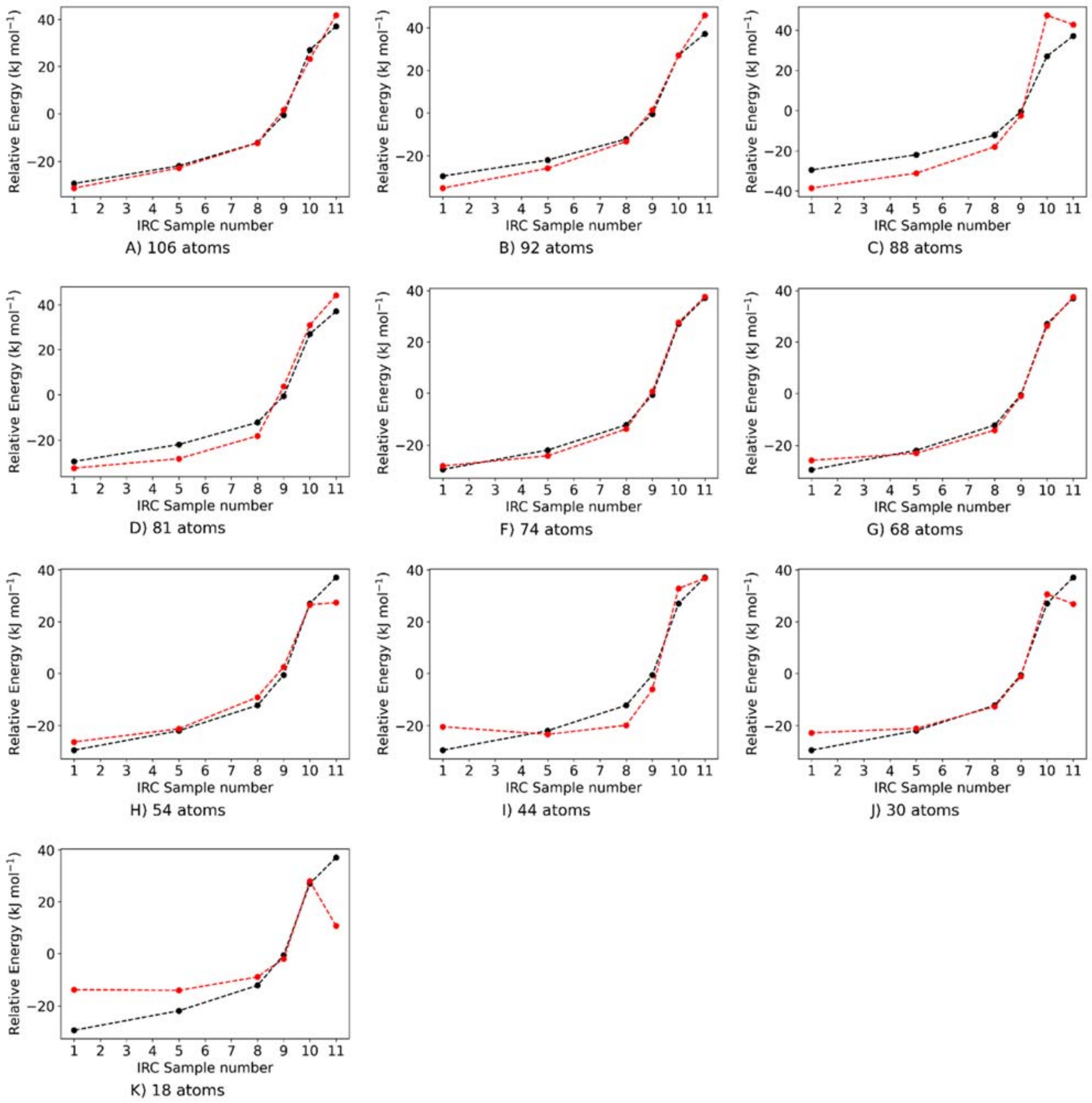

Figure S11. Linear coefficient (*m*), determination coefficient (R^2) and the ratio between the lite system energy of activation(ΔE_{lite}) and large system energy of activation (ΔE_{large}) *versus* the number of atoms at each truncation for the HIV-1 protease system.

7.1 Hydrogen Addition

As pointed out in the main text, hydrogen atoms were added at the point of truncation at a distance equal to their van der Waals radii. However, geometry optimisation of the newly formed H-A, with *A* being any atom left un-bonded from the original bond, would be intuitively needed given that the *H* atom is not at its lowest energy state due to being added at an arbitrary distance. However, does this strongly affect the energy gradients of the PES under study?

Table S7 shows a comparison of the m, R^2 and $\frac{\Delta E_{lite}}{\Delta E_{full}}$ error metrics for atom groups *G, H, I* and *J* of Figure S10 in their optimised and non-optimised states. The optimisation was performed by constraining (i.e. "freezing") all the atoms of a group at each stationary point of the PES while letting the *H* atoms added from the truncation to relax. Note that optimising the whole group is beyond the scope of this study and would lead to prolonged simulation protocols. Indeed, these geometries are a truncation of a larger system that is already optimised, and the objective is to find the optimal number of atoms that best recovers the energy gradients of that initial system. It is easily observed that the optimisation of the added hydrogen atoms has little to no impact on the error metrics; thus, the energy gradients of the PES of these truncations are almost the same in both cases, and there is no practical advantage in optimising the truncations for the following REG-IQA analysis.

Table S7. Linear coefficient (m), determination coefficient (R^2) and energy ratio ($\frac{\Delta E_{lite}}{\Delta E_{full}}$ comparison between optimised (opt) and non-optimised (non-opt) structures of groups *G, H, I* and *J* of the HIV-1 protease system.

	**opt**			**non-opt**		
**Group**	m	R^2	$\frac{\Delta E_{lite}}{\Delta E_{full}}$	m	R^2	$\frac{\Delta E_{lite}}{\Delta E_{full}}$
G	0.987	0.99	0.958	0.982	0.99	0.951
H	0.853	0.98	0.806	0.855	0.98	0.806
I	1.000	0.95	0.902	1.002	0.94	0.859
J	0.865	0.97	0.806	0.864	0.96	0.746

7.2 HIV-1 Protease 68 atoms

The REG-IQA results for the 68 atoms system of group *G* (Figure S10 (g)) are presented here. The first detail is that the number atoms that can be considered are not 68 but 64 because four hydrogens were added due to truncation. These hydrogens are not relevant to the REG-IQA analysis because they are not part of the full 133-atom system; thus, there is no need to calculate their interactions with other atoms. However, for the sake of completeness these atoms were still included in the computation, but not in the final analysis. Table S8 shows the REG-V_{xc} and REG-V_{cl} values and their ranking. These values should be compared with the ones for the full 133-atom system (Table S3). A strong agreement of both REG values and rankings is observed, thus demonstrating that using almost half of the atoms for the IQA analysis gives the same chemically relevant information. Moreover, the correlation curve in Figure S12 shows nearly perfect agreement between summed IQA energies and the original QM energies.

This approach is similar to the one shown in the main manuscript, where 27 atoms were arbitrarily picked, and the REG-IQA analysis was performed on the small wave-function. However, in this case, the wave-function at each stationary point has the same properties as the full system wave-function, while the chosen 27 atoms were not enough to fully recover the PES. Moreover, this procedure is also more accurate than the one used in the "biased" approach because the pool of IQA energies is substantially increased while maintaining computational efficiency. In that case, 27 atoms were selected, and the IQA integrations were done using the full system wave-function. That resulted in a two-day computation time per geometry using 8 CPU cores, and precise IQA energies were calculated but with discrepancies in the REG analysis due to consideration of fewer interactions. The 68-atom calculations do not recover the exact energy values, given the smaller and slightly different wave-function employed, but output a more meaningful REG analysis compared to the 27-atom system. Moreover, the computational efficiency is still maintained, if not improved, due to a smaller wave-function. Indeed, the calculations overall took ~2 CPU days per stationary point on 8 CPU cores.

Table S8. HIV protease G subset V_{xc} (left) and V_{cl} (right) REG values on points (1,5,8,9,10,11) of the original PES.

TERM	REG	R
V_{xc}(c58,o66)	-4.4	-0.91
V_{xc}(o67,h100)	-4.0	-0.97
V_{xc}(c35,o36)	-1.9	-0.97
V_{xc}(o37,h99)	-1.0	-0.99
V_{xc}(o66,o67)	-0.8	-0.99
V_{xc}(o66,h101)	1.1	0.94
V_{xc}(c35,o37)	1.5	0.96
V_{xc}(o66,h99)	1.9	0.98
V_{xc}(c58,o67)	2.5	0.97
V_{xc}(o36,h100)	3.8	0.97

TERM	REG	R
V_{cl}(o67,h100)	-7.0	-0.97
V_{cl}(c35,o36)	-5.6	-0.98
V_{cl}(c35,h100)	-3.5	-0.97
V_{cl}(c58,o66)	-2.8	-1.00
V_{cl}(o37,h99)	-2.8	-0.94
V_{cl}(c58,h100)	2.4	0.98
V_{cl}(c35,h99)	2.9	1.00
V_{cl}(o36,h100)	6.8	0.96
V_{cl}(c58,n59)	6.9	0.77
V_{cl}(c58,o67)	8.4	0.92

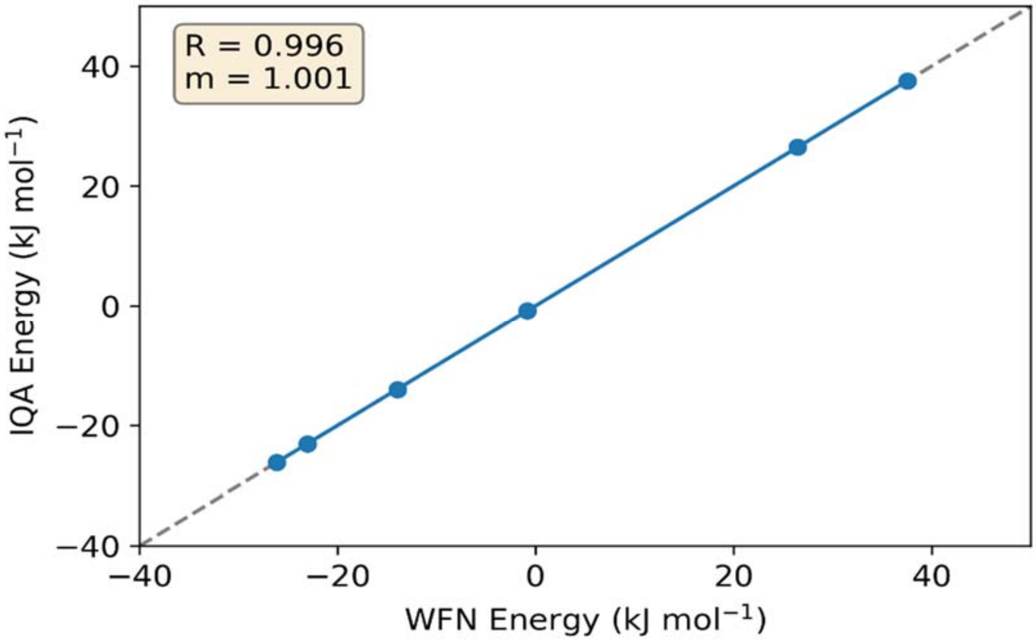

Figure S12. Correlation curve between the wave-function and IQA energies for the HIV-1 protease 68-atoms truncation.

8 IQA Timings for HIV-1 Protease

Timings for the IQA integrations (both intra- and interatomic) in the original (133 atoms wavefunction), biased (27 atoms integrated over the 133 atoms wavefunction) and unbiased (68 atoms wavefunction) analyses, as CPU hours averaged over all single-point energies. Note that the calculations were carried out on heterogeneous hardware and that each geometry was computed on 8 CPU cores. Original and unbiased calculations were also carried out with the lowest settings for the IQA integrations, which correspond to "mesh = sparse" and "outer angular quadrature = GS1" (≤ 1800 points).

	**Original (default)**	**Original (sparse)**	**Biased**	**Unbiased (default)**	**Unbiased (sparse)**
**CPU hours**	666.6	247.5	66.6	49.4	19.9
**Speed-Up**	1x	2.7x	10x	14x	33.5x
**Total elapsed average time for 11 steps (days)**	305.8	113.4	30.5	22.7	9.1
**Total elapsed average time for 6 steps with RDP (days)**	166.8	61.9	16.7	12.4	5.0

9 HheC system

9.1 RDP algorithm

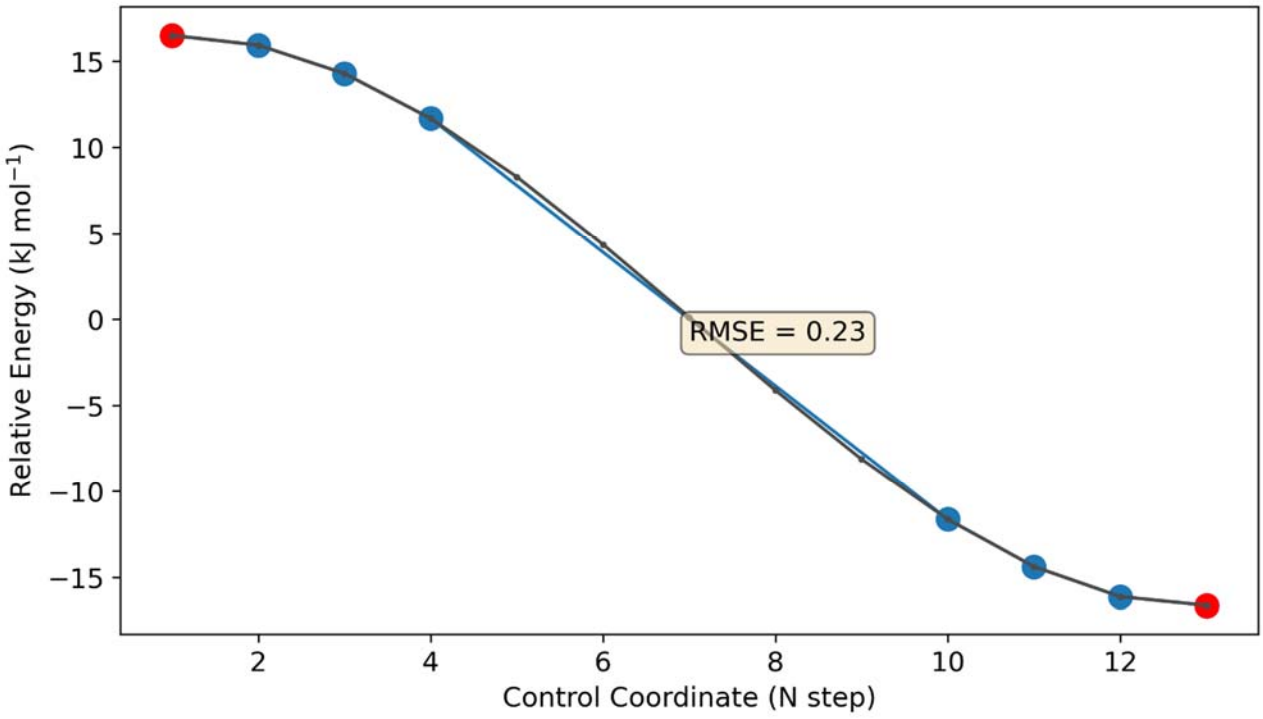

Figure S13. Ramer-Douglas-Peucker algorithm applied to the reaction coordinate of the HheC system. The transition state is on the left and the reactant on the right. The RMSE is chosen to be 0.25 kJ/mol. Points [1,2,3,4,10,11,12,13] are the points selected by the algorithm with a 0.23 kJ/mol actual RMSE. These 8 points are highlighted with small disks.

9.2 Timings

Timings (in hours) of the IQA calculations for the HheC system and truncations (average over all the 8 single points):

- Full system (112 atoms): 119 h
- 88 atoms truncation: 54.8 h
- 37 atoms truncation: 4.9 h

IQA calculations were performed with “mesh=sparse “and “outer angular quadrature=GS1” (≤ 1800 points) on 8 CPU cores.
